# Effect of cryopreservation duration on pregnancy outcomes in embryos: a systematic review and meta-analysis

**DOI:** 10.1093/hropen/hoag077

**Published:** 2026-08-13

**Authors:** Bowen Wei, Yinni Chen, Jiayi Nie, Xiating Li, Kecheng Wei, Yuxiang Wan, Jingzhen Lai

**Affiliations:** Department of Reproductive Medicine, The Reproductive Hospital of Guangxi Zhuang Autonomous Region, Nanning, China; Life Sciences Institute, Guangxi Medical University, Nanning, China; Life Sciences Institute, Guangxi Medical University, Nanning, China; School of Basic Medical Sciences, Guangxi Medical University, Nanning, China; School of Basic Medical Sciences, Guangxi Medical University, Nanning, China; Life Sciences Institute, Guangxi Medical University, Nanning, China; Life Sciences Institute, Guangxi Medical University, Nanning, China; Biobank of Guangxi Medical University, Guangxi Medical University, Nanning, China

**Keywords:** embryo, cryopreservation, duration, long-term, pregnancy

## Abstract

**STUDY QUESTION:**

Is prolonged cryopreservation associated with adverse clinical pregnancy and live birth after frozen-thawed embryo transfer?

**SUMMARY ANSWER:**

Prolonged embryo cryopreservation is associated with lower clinical pregnancy and live birth rates, particularly within the first 3–12 months of storage, but rates plateau after 2 years.

**WHAT IS KNOWN ALREADY:**

Evidence on the impact of cryopreservation duration on reproductive outcomes remains scarce and conflicting, with some studies reporting no adverse effects and others suggesting increased risks of pregnancy loss, preterm birth, and epigenetic alterations after prolonged storage.

**STUDY DESIGN, SIZE, DURATION:**

Systematic review and meta-analysis of studies comparing pregnancy outcomes by cryopreservation duration. PubMed, Embase, and the Cochrane Library were searched from inception to 31 December 2025, for English-language articles. The search strategy combined terms for embryos (e.g. ‘Embryo’, ‘Blastocyst’), cryopreservation techniques (e.g. ‘vitrification’, ‘cryopreservation’, ‘freezing’), and pregnancy outcomes (e.g. ‘pregnancy’, ‘live birth’) using Boolean operators. A total of 5208 records were identified from PubMed, 9272 from Embase, and 331 from the Cochrane Library.

**PARTICIPANTS/MATERIALS, SETTING, METHODS:**

Two independent reviewers screened studies, extracted data, and assessed quality using the Newcastle–Ottawa Scale. Inclusion required reporting of clinical pregnancy rate (CPR) or live birth rate (LBR) stratified by cryopreservation duration. Studies on donated embryos, preimplantation genetic testing, or gamete cryopreservation were excluded. Pooled odds ratios (ORs) with 95% CIs were calculated using fixed- or random-effects models based on heterogeneity (*I*²). Subgroup analyses were performed for neonatal outcomes (singleton vs. multiple births). Publication bias was assessed via funnel plots.

**MAIN RESULTS AND THE ROLE OF CHANCE:**

A total of 14 811 records were screened (5208 from PubMed, 9272 from Embase, 331 from Cochrane). After removal of duplicates, 11 077 records were screened by title and abstract, and 785 full-text articles were assessed for eligibility. Twenty-three studies (254 017 transfer cycles/patients) were included. Embryos cryopreserved >5 years had lower survival rates (OR = 0.84, 95% CI: 0.72–0.98, *P* = 0.03; *I*^2^ = 0%, *P* = 0.99). Compared with ≤3 months storage, longer storage was associated with reduced CPR (>3 months: OR = 0.87, 95% CI: 0.78–0.96, *P* = 0.005; >6 months: OR = 0.70, 95% CI: 0.66–0.74, *P* < 0.00001; >12 months: OR = 0.71, 95% CI: 0.61–0.82, *P* < 0.00001; >2 years: OR = 0.77, 95% CI: 0.65–0.91, *P* = 0.003), reduced LBR (>3 months: OR = 0.90 95% CI: 0.84–0.95, *P* = 0.0008; >6 months: OR = 0.74, 95% CI: 0.69–0.80, *P* < 0.00001; >12 months: OR = 0.72, 95% CI: 0.63–0.84, *P* < 0.0001; >2 years: OR = 0.80, 95% CI: 0.72–0.89, *P* < 0.0001) and reduced biochemical pregnancy rate (>3 months: OR = 0.82, 95% CI: 0.73–0.92, *P* = 0.0007; >6 months: OR = 0.70, 95% CI: 0.63–0.77, *P* < 0.00001; >12 months: OR = 0.72, 95% CI: 0.65–0.80, *P* < 0.00001; >2 years: OR = 0.74, 95% CI: 0.61–0.90, *P* = 0.002). Miscarriage rate (OR = 1.16, 95% CI: 1.09–1.25, *P* < 0.0001) and preterm birth rate (OR = 1.10, 95% CI: 1.00–1.20, *P* = 0.04) were higher after >12 months storage. High birth weight and malformation rates were not significantly increased, but low birth weight was less frequent after >6 (OR = 0.83, 95% CI: 0.70–0.99, *P* = 0.04) or >12 months (OR = 0.75, 95% CI: 0.57–0.98, *P* = 0.03), and the proportion of male infants was lower after >3 months (OR = 0.92, 95% CI: 0.86–0.98, *P* = 0.02) and >2 years (OR = 0.79, 95% CI: 0.64–0.97, *P* = 0.02), compared with those cryopreserved for ≤3 months. No significant publication bias was detected.

**LIMITATIONS, REASONS FOR CAUTION:**

Limitations include substantial heterogeneity across studies (design, cryopreservation method, embryonic stage, confounder adjustment), inability to evaluate frozen-thawed embryo transfer (FET) cycle type, maternal age, and embryo quality due to inconsistent reporting, retrospective observational design precluding causal inference, residual confounding, potential survivor bias, and combined singleton/multiple-birth data for neonatal outcomes. Despite a large sample size (254 017 cycles), these factors warrant cautious interpretation.

**WIDER IMPLICATIONS OF THE FINDINGS:**

This comprehensive meta‑analysis reveals a non‑linear relationship between cryopreservation duration and outcomes, reconciling previous conflicting reports by demonstrating early declines (first 3–12 months) followed by a plateau beyond 2 years. The stability of outcomes in ultra‑long storage is reassuring for patients requiring delayed transfer. These findings support clinical counselling that encourages early transfer when feasible, but provides reassurance that ultra‑long storage does not lead to progressive deterioration in pregnancy or neonatal outcomes.

**FUNDING:**

This work was funded by the First-class discipline innovation-driven talent program of Guangxi Medical University (to J.L.) and the College Students’ Innovation Training Project of Guangxi Medical University (X202410598348).

**DISCLOSURES:**

The authors declare no conflicts of interest.

**REGISTRATION NUMBER:**

CRD42024555362.

WHAT DOES THIS MEAN FOR PATIENTS? When people undergo fertility treatment, any remaining embryos can be frozen (cryopreserved) for future use. This is common practice, but until now it has not been clear whether storing embryos for longer periods affects the chances of a successful pregnancy.

In this study, we reviewed 23 research papers involving over 250 000 embryo transfer cycles to see if the length of storage time made a difference. We found that embryos that had been frozen for more than 5 years were slightly less likely to survive the thawing process. Compared with embryos stored for 3 months or less, those stored for longer than 3 months (and especially beyond 6 or 12 months) had lower rates of pregnancy and live birth. The risk of miscarriage was also higher after 12 months of storage. However, after 2 years of storage, the outcomes stopped getting worse—they stayed the same even with much longer storage. On a positive note, longer storage did not increase the risk of premature birth, high birth weight, or birth defects. One interesting finding was that when embryos were stored for more than 3 months, the chance of having a male baby was slightly lower.

Therefore, using frozen embryos sooner (within the first few months), if possible, may give the best chance of pregnancy and live birth. But if longer storage is needed, this study suggests that after 2 years the risks do not continue to increase. Ultra-long storage (beyond 2 years) does not appear to cause further harm. These findings can help doctors and patients make more informed decisions about when to use frozen embryos.

## Introduction

Since the first use of frozen-thawed embryo transfer (FET) in 1983 (Ludwig *et al.*, 1999), it has become an integral component of ART. Accumulated evidence indicates that cryopreservation confers several advantages. It allows utilization of supernumerary embryos generated during fresh IVF cycles, thereby improving cumulative live birth rates (LBR) and reducing costs, mitigates the risk of ovarian hyperstimulation syndrome (OHSS), and facilitates fertility preservation in patients with autoimmune disorders or malignancies (Devroey *et al.*, 2011; Zhang *et al.*, 2018; Ahuja and Macklon, 2020).

Continuous refinement of cryopreservation protocols has led to a rapid expansion in both the number of cryopreserved embryos and FET cycles (Yang *et al.*, 2022). Data published by ESHRE and the American Society for Reproductive Medicine (ASRM) demonstrate a year-on-year increase in the proportion of FET cycles performed (Sunderam *et al.*, 2022; Wyns *et al.*, 2022). Nevertheless, concerns persist regarding the long-term safety of cryopreservation and the potential impact of cryopreservation duration. The two principal technologies currently in use are slow freezing and vitrification. Over the past decades, vitrification has largely superseded slow freezing and is now the preferred method for embryo cryopreservation (Nagy *et al.*, 2020). Vitrification solidifies the intra- and extracellular milieu into an amorphous ‘glass’, thereby restricting macromolecular diffusion. However, localized micro-movement of solutes may still occur at or below the glass-transition temperature (*T*_g_) (Fahy and Wowk, 2021). In addition, the safety implications of high-concentration cryoprotectants and open-system storage, where embryos are in direct contact with liquid nitrogen, remain incompletely characterized (Bielanski, 2014; Molina *et al.*, 2016).

Evidence on the impact of cryopreservation duration on reproductive outcomes remains scarce and conflicting. Several studies have reported that length of cryopreservation did not compromise clinical outcomes after embryo transfer. A retrospective cohort reported on 3367 vitrified–warmed embryo transfers stratified into cryopreservation durations of 12–23 months, 24–35 months, 36–48 months and ≥48 months and observed no significant differences in embryo implantation rate (IR), clinical pregnancy rate (CPR), singleton birth weight or LBR among groups (Liu *et al.*, 2014). Likewise, a study evaluating storage duration (maximum 160 months) for slow-frozen early-cleavage embryos found comparable CPR and LBR, as well as analogous neonatal sex ratio, singleton gestational age, singleton birth weight and malformation rate between different cryopreservation duration groups, indicating no clinically relevant effect of prolonged cryopreservation (Shi *et al.*, 2022). Moreover, the duration of embryo cryopreservation showed no demonstrable association with the incidence of pregnancy-associated complications such as gestational diabetes mellitus or hypertensive disorders of pregnancy (Xu *et al.*, 2022).

Nevertheless, a growing number of studies have reported that FET may influence the incidence of maternal obstetric complications, pregnancy outcomes, neonatal malformations, and even the long-term health of offspring. Some studies have observed an increased risk of pregnancy-related complications following FET, such as gestational hypertension and postpartum hemorrhage (Al-Khatib *et al.*, 2024; Yan *et al.*, 2026). Hu *et al.* (2022) described a non-linear (inverted U-shaped) relationship between embryo cryopreservation duration and FET success in women with high ovarian response, with prolonged cryopreservation associated with reduced pregnancy rates. It has been reported that cryopreservation exceeding 1 year is associated with an increased risk of early pregnancy loss and preterm birth (Wang *et al.*, 2024b). When cryopreservation duration surpasses 5 years, significant declines in embryo IR and LBR have been reported (Zhan *et al.*, 2024). Moreover, cryopreservation beyond 6 years was reported to be associated with a significantly higher incidence of ectopic pregnancy (Wang *et al.*, 2024b). Although population-based studies have not reported an increased prevalence of birth defects following FET cycles, animal studies have demonstrated that vitrification and cryoinjury can alter gene expression and embryonic epigenetic profiles (Li *et al.*, 2021; Wang *et al.*, 2024a; Chen *et al.*, 2025b). Multi-omic analyses reveal that these alterations predominantly involve pathways related to nervous system development, cardiovascular function, and glucose–lipid metabolism, suggesting that cryopreservation-induced epigenetic changes may have long-term implications for offspring health (Chen *et al.*, 2020). Collectively, current evidence remains insufficient to draw definitive conclusions regarding the impact of cryopreservation, particularly prolonged cryopreservation, on the clinical outcomes of embryo transfer.

In aggregate, the literature remains divided as to whether prolonged cryopreservation compromises the reproductive performance of FET. To clarify the putative consequences of prolonged cryopreservation, we therefore conducted a systematic review and meta-analysis of all available data, comparing the influence of cryopreservation duration on a comprehensive spectrum of post-transfer endpoints. The resultant evidence is intended to inform best-practice decisions regarding the prolonged banking of human embryos and the subsequent clinical utilization of such long-term cryopreserved material.

## Materials and methods

### Search criteria

On 31 December 2025, studies were screened independently by two investigators (Y.C. and J.N.) from various databases, including PubMed, Embase, and Cochrane Library (Cochrane Database of Systematic Reviews, Cochrane Central Register of Controlled Trials, and Cochrane Methodology Register), with no restriction on time (see Supplementary Table S1 for the detailed search strategy). All studies were published in English. This study was registered in the International Prospective Register of Systematic Reviews (PROSPERO ID: CRD42024555362).

### Inclusion and exclusion criteria

Articles were included if they compared the pregnancy outcomes of groups differentiated by cryopreservation duration. In addition, the included studies had at least one of the following quantitative outcomes: CPR and LBR. Studies referring to donated embryos, preimplantation genetic testing, and sperm/oocyte cryopreservation were not included. Additionally, review articles, editorials, letters to the editor, case reports, abstracts from conferences, and animal experimental studies were excluded.

### Study selection and data extraction

Two reviewers (among Y.C., J.N., X.L., K.W., and Y.W.) independently screened each title and abstract of all retrieved articles. Any disagreements between reviewers were settled by consensus with involvement of a third reviewer (J.L. or B.W.) as needed. If relevant, full texts were then reviewed to identify eligible studies based on the inclusion and exclusion criteria. For each included study, two reviewers (among Y.C., J.N., X.L., K.W., and Y.W.) extracted data into an Excel template. A third reviewer (J.L. or B.W.) verified all extracted data, and disagreements were handled through consensus.

Data retrieved from the articles included first author, publication year, location, study design, time period, method of embryo cryopreservation, cryopreservation duration groups, number of embryos, number of cycles/patients, and outcomes. Outcomes referring to embryo viability, pregnancy outcomes, and neonatal outcomes were evaluated. Among these, embryo viability was mainly characterized by embryo survival rate (SR) (the number of survived embryos divided by the number of thawed embryos) and embryo IR (the number of gestational sacs observed using ultrasound per transferred embryo). Pregnancy outcomes included CPR (clinical pregnancy [the presence of an intrauterine gestational sac at 6 weeks of gestation in the transvaginal ultrasound] per embryo transfer cycle), LBR (number of live births of at least one infant per embryo transfer cycle), biochemical pregnancy rate (BPR) (number of serum HCG positivity 10 to 14 days after transfer per embryo transfer cycle), ectopic pregnancy rate (EPR) (number of at least one extrauterine cavity gestational sac per embryo transfer cycle), and miscarriage rate (MR) (the number of clinical pregnancy losses per clinical pregnancy). The preterm birth rate (PBR) (the number of newborns with <37 weeks’ gestation per all newborns), low birth weight rate (LBWR) (the number of newborns with birth weight <2500 g per all newborns), high birth weight rate (HBWR) (the number of newborns with birth weight >4000 g per all newborns), congenital malformation rate (CMR, the number of newborns with congenital malformation per all newborns) and male children rate (the number of male newborns per all newborns) were selected to describe neonatal outcomes.

### Quality assessment

The quality of the studies was evaluated using the Newcastle–Ottawa scale (NOS) (Wells *et al.*, 2025) by the same reviewer who extracted data. The studies reaching a score of 7 were considered to be of high quality, while studies with a total score <5 were assessed as having low quality. Disagreements between reviewers were resolved by discussion.

### Statistical analysis

In the process of data consolidation, we standardized the expression of time. For example, cryopreservation duration of 30 days, 4 weeks, and 1 month were all unified as 1 month; cryopreservation duration of 60 days, 8 weeks, and 2 months were all unified as 2 months; cryopreservation duration of 90 days, 12 weeks, and 3 months were all unified as 3 months; cryopreservation duration of 360 days, 365 days, 52 weeks, 12 months and 1 year were all unified as 12 months; and so on. Since different studies may have time grouping settings that include or exclude the upper and lower limits of the groups, to facilitate statistical data analysis, we did not specifically distinguish whether the upper and lower limits of the groups were included, and assumed that all groups included the lower limit but not the upper limit. For example, for a cryopreservation duration group of 3 months, it was divided into groups of ≤3 months and >3 months. Based on the time grouping of the included studies, the current meta-analysis grouped the cryopreservation time of embryos at intervals of 3 months, 6 months, 12 months, 2 years, 3 years, and 5 years to compare the differences in outcomes.

Odds ratios (ORs) with 95% CIs were chosen to analyze dichotomous variables. Heterogeneity was quantified by the *I*^2^ statistic. The *I^2^* statistic quantifies the percentage of total variation across studies due to true between-study differences rather than chance. According to the Cochrane Handbook of Systematic Review, *I*^2^ values of 0–40%, 30–60%, 50–90%, and 75–100% represent unimportant, moderate, substantial, and considerable heterogeneity, respectively (Higgins *et al.*, 2024). We used a 50% threshold to select between fixed-effects and random-effects models, following standard Cochrane practice. A random-effects model was used if *I*^2^ was >50%; otherwise, the fixed-effects model was used. Subgroups were created, with singleton newborns or multiple newborns, to reduce the influence on neonatal outcomes. Sensitivity analyses were performed by removing one study at a time to evaluate the quality and consistency of the meta-analysis results. Publication bias was screened for using funnel plots. All *P*-values were two-sided, and a *P*-value < 0.05 was considered statistically significant. All statistical analyses were conducted using Review Manager (version 5.3, Cochrane Collaboration, Oxford, UK).

## Results

### Study selection and characteristics of included studies

**Figure 1.**
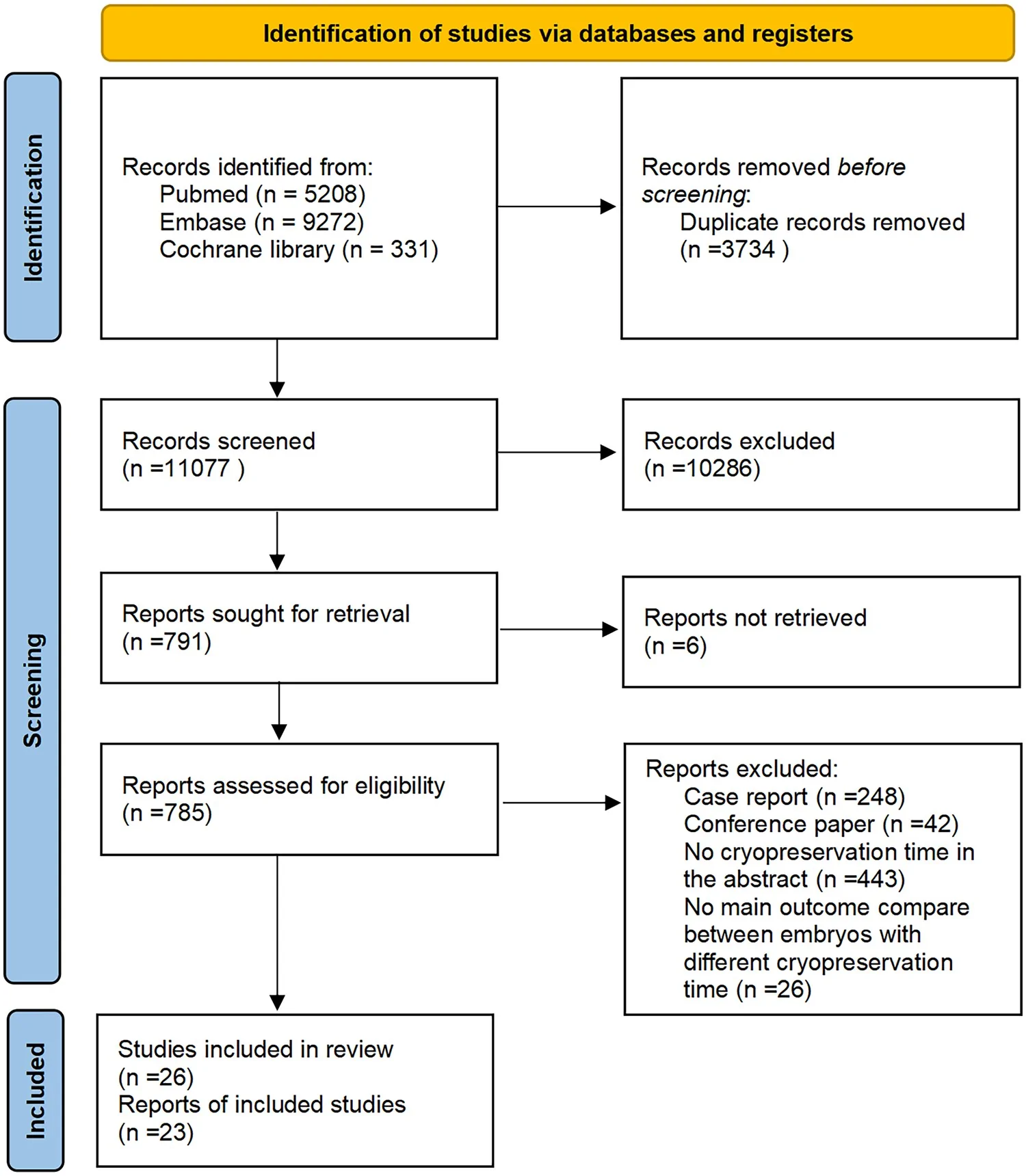
**Flow chart of search and selection strategy**.

A literature search of PubMed, Embase, and the Cochrane Library (Cochrane Database of Systematic Reviews, Cochrane Central Register of Controlled Trials, and Cochrane Methodology Register) yielded 5208, 9272, and 331 records, respectively. After the screening procedure outlined in Fig. 1, 26 publications published between 1988 and 2025 were retained for full-text review, including 25 retrospective cohort studies and one investigation whose design was not explicitly stated. One article (Cohen *et al.*, 1988) was excluded from the meta-analysis because its recruitment era predated contemporary cryopreservation protocols, and the cryopreservation time-interval categories did not overlap with those of the remaining studies. Another two articles (He *et al.*, 2023; Chen *et al.*, 2025a) were likewise excluded because inter-group comparisons were performed after propensity-score matching, a design feature incompatible with the crude effect estimates reported by the other studies. Consequently, 23 studies, originating from Australia, China, Japan, South Korea, India, and Brazil, were pooled, encompassing 254 017 cryopreserved embryo transfer cycles/patients included in the final meta-analysis (Table 1). As evaluated using Newcastle–Ottawa Scale guidelines, the quality of the included studies was assessed to be medium or high (Table 2).

**Table 1.** Characteristics of included studies and participants.

Study	Location	Study design	Time period	Method of embryo cryopreservation	Cryopreservation duration groups	Number of embryos	Number of cycles/patients	Outcomes assessed
Wirleitner *et al.* (2013)	Austria	Retrospective study	January 2009–April 2012	Vitrification	Group 1: 0–3 months Group 2: 3–6 months Group 3: 6–12 months Group 4: 12–24 months Group 5: 24–36 months Group 6: 36–48 months Group 7: 48–72 months	Group 1: 341 Group 2: 543 Group 3: 325 Group 4: 305 Group 5: 297 Group 6: 98 Group 7: 83	Group 1: 100 Group 2: 169 Group 3: 99 Group 4: 92 Group 5: 90 Group 6: 27 Group 7:26	Embryo survival, embryo implantation, biochemical pregnancy, clinical pregnancy, live birth, congenital malformation
Liu *et al.* (2014)	China	Retrospective study	January 2005–March 2012	Slow freezing	Group 1: 12–23 months Group 2: 24–35 months Group 3: 36–47 months Group 4: > 48 months	Group 1: 1393 Group 2: 1132 Group 3: 421 Group 4: 421	Group 1: 360 Group 2: 291 Group 3: 112 Group 4: 104	Embryo survival, embryo implantation, clinical pregnancy, live birth, low birth weight
Li *et al.* (2017)	China	Retrospective study	January 2013–October 2013	Vitrification	Group 1: 1–3 months Group 2: 4–6 months Group 3: 7–12 months Group 4: 13–24 months Group 5: 25–60 months	Group 1: 290 Group 2: 626 Group 3: 452 Group 4: 249 Group 5: 122	Group 1: 147 Group 2: 295 Group 3: 195 Group 4: 97 Group 5: 46	Embryo survival, embryo implantation, clinical pregnancy, live birth, congenital malformation, newborn gender
Ueno *et al.* (2018)	Japan	Retrospective cohort study	January 2007–December 2015	Vitrification	Group 1: 0–2 months Group 2: 2–13 months Group 3: 13–97 months	Group 1: 4702 Group 2: 2853 Group 3: 1181	Group 1: 4702 Group 2: 2853 Group 3: 1181	Embryo survival, clinical pregnancy, miscarriage, live birth, congenital malformation, newborn gender
Li *et al.* (2020)	China	Retrospective study	January 2011–December 2017	Vitrification	Group 1: < 3 months Group 2: 3–6 months Group 3: 6–12 months Group 4: 12–24 months	Group 1: 3377 Group 2: 2705 Group 3: 814 Group 4: 110	Group 1: 3377 Group 2: 2705 Group 3: 814 Group 4: 110	Embryo survival, preterm birth, low birth weight, high birth weigh, congenital malformation, newborn gender
Cui *et al.* (2021)	China	Retrospective cohort study	January 2016–September 2019	Vitrification	Group 1: < 3 months Group 2: 3–12 months Group 3: > 12 months	Group 1: 4892 Group 2: 3499 Group 3: 1415	Group 1: 4892 Group 2: 3499 Group 3: 1415	Embryo survival, clinical pregnancy, live birth, ectopic pregnancy, miscarriage, preterm birth, congenital malformation, newborn gender
Lee *et al.* (2021)	Korea	Retrospective cohort study	January 2013–June 2014	Vitrification	Group 1: 0–6 months Group 2: 7–12 months Group 3: 13–24 months Group 4: ≥ 25 months	Group 1: 1642 Group 2: 534 Group 3: 289 Group 4: 403	Group 1: 937 Group 2: 299 Group 3: 165 Group 4: 231	Embryo survival, embryo implantation, clinical pregnancy, live birth, miscarriage, preterm birth, low birth weight, high birth weight, congenital malformation, newborn gender
Lin *et al.* (2021)	China	Retrospective study	January 2006–December 2017	Vitrification	Group 1: 28 days—1 year Group 2: 1–3 years Group 3: 3–5 years Group 4: ≥ 5 years	Group 1: 5626 Group 2: 1467 Group 3: 646 Group 4: 187	Group 1: 5369 Group 2: 1401 Group 3: 624 Group 4: 185	Embryo survival, clinical pregnancy, live birth, ectopic pregnancy, miscarriage
Zhang *et al.* (2021)	China	Multicenter retrospective study	January 2014–December 2018	Vitrification	Group 1: 3–8 weeks Group 2: 8–12 weeks Group 3: 12–26 weeks Group 4: 26–52 weeks Group 5: > 52 weeks	N/A	Group 1: 4734 Group 2: 6779 Group 3: 5028 Group 4: 958 Group 5: 327	Biochemical pregnancy, clinical pregnancy, live birth, ectopic pregnancy, miscarriage
Hu *et al.* (2022)	China	Retrospective cohort study	January 2013–December 2019	Vitrification	Group 1: 0.8–1.0 months Group 2: 1.1–2.0 months Group 3: 2.1–3.0 months Group 4: 3.1–4.0 months Group 5: 4.1–5.0 months Group 6: 5.1–6.0 months Group 7: 6.1–12.0 months Group 8: > 12.0 months	N/A	Group 1: 403 Group 2: 1855 Group 3: 6652 Group 4: 2932 Group 5: 1308 Group 6: 624 Group 7: 884 Group 8: 270	Biochemical pregnancy, clinical pregnancy, live birth, miscarriage
Mao *et al.* (2022)	China	Retrospective study	January 2004–August 2019	Vitrification	Group 1: 1–90 days Group 2: 91–180 days Group 3: 181–365 days Group 4: 366–730 days Group 5: > 731 days	Group 1: 35 338 Group 2: 12 104 Group 3: 4656 Group 4: 1626 Group 5: 1801	Group 1: 20 926 Group 2: 6472 Group 3: 2237 Group 4: 746 Group 5: 762	Embryo survival, embryo implantation, biochemical pregnancy, clinical pregnancy, live birth, ectopic pregnancy, low birth weight, high birth weight, congenital malformation, newborn gender
Reddy *et al.* (2022)	India	Retrospective cohort study	January 2012–December 2020	Vitrification	Group 1: 3–5 years Group 2: > 5 years	Group 1: 159 Group 2: 43	Group 1: 75 Group 2: 20	Embryo survival, embryo implantation, biochemical pregnancy, clinical pregnancy, live birth, miscarriage
Shi *et al.* (2022)	China	Retrospective cohort study	February 2013–August 2017	Slow-freezing	Group 1: 6–12 months Group 2: 13–36 months Group 3: 37–60 months Group 4: 61–84 months Group 5: > 84 months	Group 1: 2130 Group 2: 986 Group 3: 631 Group 4: 558 Group 5: 325	Group 1: 770 Group 2: 359 Group 3: 220 Group 4: 177 Group 5: 98	Embryo survival, embryo implantation, clinical pregnancy, live birth, congenital malformation, newborn gender
Xu *et al.* (2022)	China	Retrospective cohort study	May 2010–September 2017	Vitrification	Group 1: ≤ 3 months Group 2: 4–6 months Group 3: 7–12 months Group 4: > 12 months	N/A	Group 1: 1549 Group 2: 1079 Group 3: 661 Group 4: 575	Preterm birth, low birth weight, high birth weight, newborn gender
Li *et al.* (2023)	China	Retrospective study	January 2012–December 2021	Vitrification	Group 1: 1–6 months Group 2: 7–12 months Group 3: 13–36 months Group 4: 37–84 months	Group 1: 612 Group 2: 202 Group 3: 141 Group 4: 76	Group 1: 612 Group 2: 202 Group 3: 141 Group 4: 76	Embryo implantation, clinical pregnancy, live birth, miscarriage, ectopic pregnancy, preterm birth, low birth weight, high birth weight
Ma *et al.* (2023)	China	Retrospective cohort study	January 2013–June 2020	Vitrification	Group 1: ≤ 3 months Group 2: 4–6 months Group 3: 7–12 months Group 4:13–24 months Group 5: 25–98 months	Group 1: 1679 Group 2: 688 Group 3: 244 Group 4: 123 Group 5: 528	Group 1: 1621 Group 2: 657 Group 3: 225 Group 4: 104 Group 5: 331	Embryo survival, biochemical pregnancy, clinical pregnancy, live birth, miscarriage, ectopic pregnancy, preterm birth, low birth weight, high birth weight
Zheng *et al.* (2023)	China	Retrospective cohort study	January 2015–December 2020	Vitrification	Group 1: 0–3 months Group 2: 4–6 months Group 3: 7–12 months Group 4:13–24 months Group 5: 25–72 months Group 6: 73–120 months	N/A	Group 1: 4309 Group 2: 1061 Group 3: 304 Group 4:113 Group 5: 466 Group 6: 74	Biochemical pregnancy, clinical pregnancy, live birth
Liang *et al.* (2024)	China	Retrospective cohort study	January 2013–December 2019	Vitrification	Group 1: 0.8–3 months Group 2: 3.1–6 months Group 3: 6.1–12 months Group 4: > 12 months	N/A	Group 1: 4504 Group 2: 3982 Group 3: 1227 Group 4: 454	Biochemical pregnancy, clinical pregnancy, live birth, miscarriage
Wang *et al.* (2024b)	China	Retrospective study	January 2015–December 2021	Vitrification	Group 1: ≤ 1 year Group 2: 1–6 years Group 3: ≥ 6 years	N/A	Group 1: 40 461 Group 2: 6337 Group 3: 208	Biochemical pregnancy, clinical pregnancy, live birth, miscarriage, ectopic pregnancy, preterm birth, newborn gender
Zhan *et al.* (2024)	China	Retrospective cohort study	January 2016–December 2022	Vitrification	Group 1: 0–2 years Group 2: 2–5 years Group 3: > 5 years	Group 1: 50 036 Group 2: 8012 Group 3: 1358	Group 1: 31 565 Group 2: 4458 Group 3: 642	embryo survival, Embryo implantation, live birth, miscarriage, ectopic pregnancy, preterm birth, congenital malformation, newborn gender
Costa *et al.* (2025)	Brazil	Retrospective study	January 2015–December 2019	Vitrification	Group 1: 1–90 days Group 2: 91–180 days Group 3: 181–360 days Group 4: > 360 days	N/A	Group 1: 844 Group 2: 304 Group 3: 172 Group 4: 248	Clinical pregnancy
Guo *et al.* (2025)	China	Retrospective cohort study	January 2016–December 2023	Vitrification	Group 1: ≤ 3 months Group 2: 4–6 months Group 3: 7–12 months Group 4:> 12 months	Group 1: 4634 Group 2: 5348 Group 3: 2356 Group 4: 2044	Group 1: 3030 Group 2: 3193 Group 3: 1465 Group 4: 1300	Clinical pregnancy, live birth, miscarriage, preterm birth
Zhao *et al.* (2026) ^i^	China	Multicenter retrospective study	March 2013–December 2018	Vitrification	Group 1: < 1 month Group 2: 1.1–2.0 months Group 3: 2.1–3.0 months Group 4: 3.1–6.0 months Group 5: 6.1–9.0 months Group 6: 9.1–12.0 months Group 7: > 12 months	Group 1: 2709 Group 2: 7393 Group 3: 20 322 Group 4: 18 133 Group 5: 1768 Group 6: 503 Group 7: 497	Group 1: 1852 Group 2: 4815 Group 3: 12 909 Group 4: 11 476 Group 5: 1134 Group 6: 325 Group 7: 327	Clinical pregnancy, live birth, miscarriage

i This study was published online ahead of print in 2025 and in print in 2026.

**Table 2.** **The quality of the included studies assessed by the Newcastle**–**Ottawa Scale.**

Study	Selection				Comparability	Outcome			Total^i^
	Representativeness of exposed cohort	Selection of the non-exposed cohort	Ascertainment of exposure	Demonstration that outcome of interest was not present at start of study	Cohorts on the basis of the design or analysis	Ascertainment of outcome	Was follow-up long enough for outcomes to occur?	Adequacy of follow-up of cohorts	
Wirleitner *et al.* (2013)	*^ii^	*	*		*	*	*		6
Liu *et al.* (2014)	*	*	*		**	*	*		7
Li *et al.* (2017)	*	*	*		*	*	*		6
Ueno *et al.* (2018)	*	*	*		*	*	*	*	7
Li *et al.* (2020)	*	*	*			*	*	*	6
Cui *et al.* (2021)	*	*	*		*	*	*	*	7
Lee *et al.* (2021)	*	*	*		*	*	*		6
Lin *et al.* (2021)	*	*	*			*	*	*	6
Zhang *et al.* (2021)	*	*	*		*	*	*		6
Hu *et al.* (2022)	*	*	*		*	*	*	*	7
Mao *et al.* (2022)	*	*	*		*	*	*	*	7
Reddy *et al.* (2022)	*	*	*			*	*	*	6
Shi *et al.* (2022)	*	*	*		*	*	*		6
Xu *et al.* (2022)	*	*	*		**	*	*	*	8
Li *et al.* (2023)	*	*	*		*	*	*		6
Ma *et al.* (2023)	*	*	*		*	*	*	*	7
Zheng *et al.* (2023)	*	*	*		*	*	*	*	7
Liang *et al.* (2024)	*	*	*		*	*	*	*	7
Wang *et al.* (2024b)	*	*	*		*	*	*	*	7
Zhan *et al.* (2024)	*	*	*		*	*	*	*	7
Costa *et al.* (2025)	*	*	*		*	*	*	*	7
Guo *et al.* (2025)	*	*	*		*	*	*	*	7
Zhao *et al.* (2026)	*	*	*		*	*	*	*	7

i The studies reaching a score of 7 were considered to be of high quality, while studies with a total score <5 were assessed as having low quality.

ii A study can be awarded a maximum of one star for each numbered item within the Selection and Exposure categories. A maximum of two stars can be given for Comparability.

### Outcomes

#### The comparison of embryo survival between different cryopreservation durations

**Figure 2.**
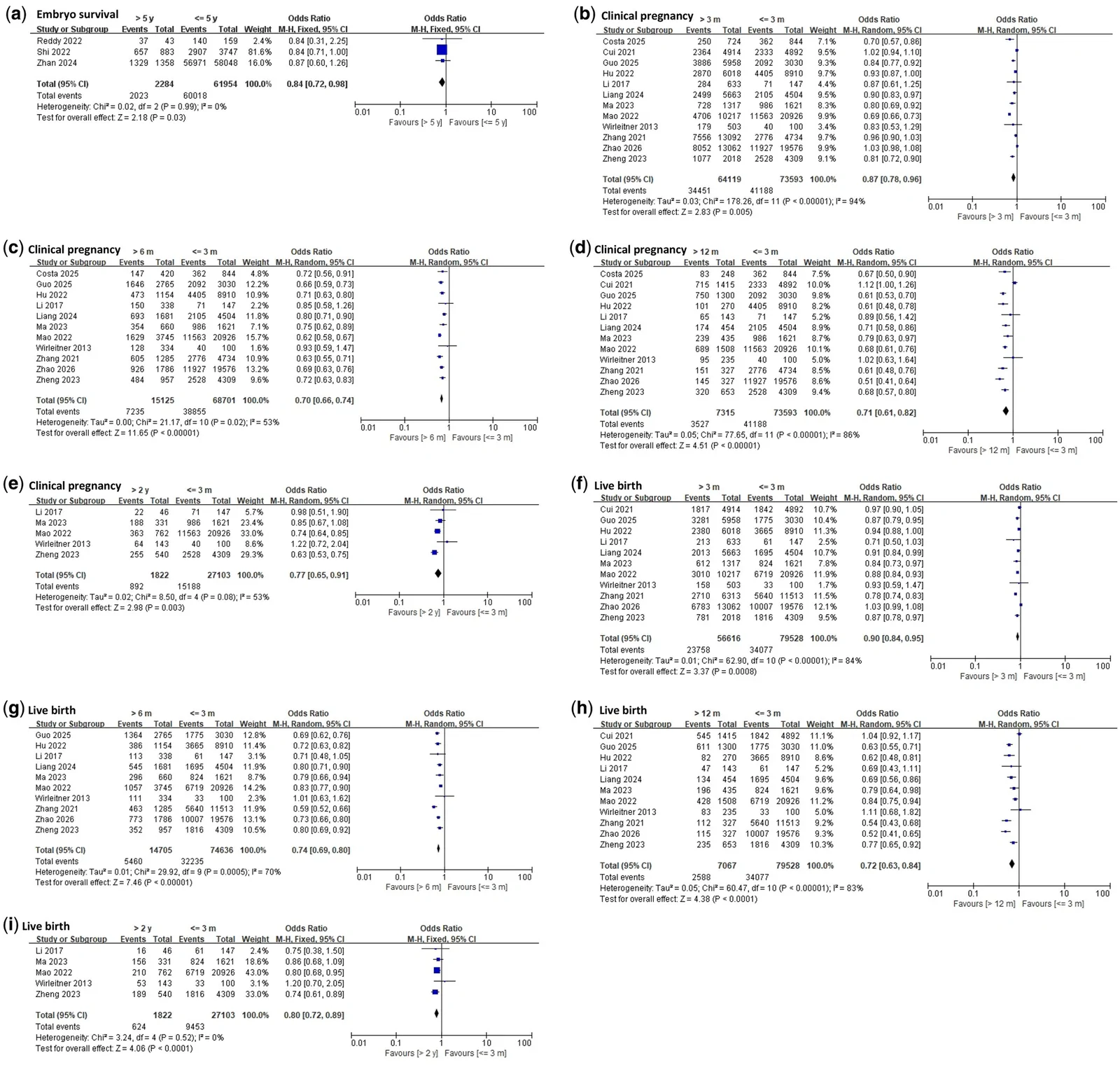
**Main comparisons of pregnancy outcomes between different cryopreservation durations.** (**a**) Embryo survival comparison between embryos cryopreserved for above 5 years or less, (**b**–**e**) clinical pregnancy comparison between embryos cryopreserved for >3 m (months) v.s. ≤ 3 m, > 6 m v.s. ≤ 3 m, >12 m v.s. ≤ 3 m, and >2 y (years) v.s. ≤ 3 m, (**f**–**i**) live birth comparison between embryos cryopreserved for > 3 m v.s. ≤ 3 m, > 6 m v.s. ≤ 3 m, > 12 m v.s. ≤ 3 m, and > 2 y v.s. ≤ 3 m. M-H: Mantel-Haenszel method.

The embryo SR was reported in 13 publications (Wirleitner *et al.*, 2013; Liu *et al.*, 2014; Li *et al.*, 2017, 2020; Ueno *et al.*, 2018; Cui *et al.*, 2021; Lee *et al.*, 2021; Lin *et al.*, 2021; Mao *et al.*, 2022; Reddy *et al.*, 2022; Shi *et al.*, 2022; Ma *et al.*, 2023; Zhan *et al.*, 2024). Cryopreservation intervals of 3 months, 6 months, 12 months, 2 years, 3 years, and 5 years were used to stratify and compare embryo survival. Embryos cryopreserved for >5 years exhibited a lower survival rate than those cryopreserved for ≤5 years (OR = 0.84, 95% CI: 0.72–0.98, *P* = 0.03; *I*^2^ = 0%, *P* = 0.99) (Fig. 2a). In contrast, when the cohorts were stratified at the remaining time-points, duration of cryopreservation had no discernible effect on embryo survival (Supplementary Fig. S1).

#### The comparison of pregnancy outcomes between different cryopreservation durations

The pregnancy outcomes were reported in 21 publications (Wirleitner *et al.*, 2013; Liu *et al.*, 2014; Li *et al.*, 2017, 2023; Ueno *et al.*, 2018; Cui *et al.*, 2021; Lee *et al.*, 2021; Lin *et al.*, 2021; Zhang *et al.*, 2021; Hu *et al.*, 2022; Mao *et al.*, 2022; Reddy *et al.*, 2022; Shi *et al.*, 2022; Ma *et al.*, 2023; Zheng *et al.*, 2023; Liang *et al.*, 2024; Zhan *et al.*, 2024; Wang *et al.*, 2024b; Costa *et al.*, 2025; Guo *et al.*, 2025; Zhao *et al.*, 2026). We then performed pooled analysis on pregnancy outcomes, including clinical pregnancy and live birth as the primary outcomes, and biochemical pregnancy, embryo implantation, ectopic pregnancy, and miscarriage as the secondary outcomes.

Pooled meta-analyses of clinical pregnancy rates are presented in Fig. 2b–e and Supplementary Fig. S2. Compared with embryos cryopreserved for ≤3 months, those cryopreserved for >3 months exhibited a significantly lower CPR (OR = 0.87, 95% CI: 0.78–0.96, *P* = 0.005; *I*^2^ = 94%, *P* < 0.0001) (Fig. 2b). This reduction was consistently observed when the cryopreservation duration threshold was advanced to >6 months (OR = 0.70, 95% CI: 0.66–0.74, *P* < 0.00001; *I*^2^ = 53%, *P* < 0.0001), >12 months (OR = 0.71, 95% CI: 0.61–0.82, *P* < 0.00001; *I*^2^ = 86%, *P* < 0.0001), or >2 years (OR = 0.77, 95% CI: 0.65–0.91, *P* = 0.003; *I*^2^ = 53%, *P* = 0.08) (Fig. 2c–e). Irrespective of whether the cut-off of the cryopreservation duration was set at 3 months (OR = 0.87, 95% CI: 0.78–0.96, *P* = 0.005; *I*^2^ = 94%, *P* < 0.0001), 6 months (OR = 0.83, 95% CI: 0.71–0.97, *P* = 0.02; *I*^2^ = 94%, *P* < 0.0001), or 12 months (OR = 0.80, 95% CI: 0.73–0.88, *P* < 0.00001; *I*^2^ = 85%, *P* < 0.0001), prolonged cryopreservation remained associated with a lower CPR (Fig. 2b and Supplementary Fig. S2a and e). However, CPR was comparable between embryos cryopreserved for ≤6 months (or any longer time) and those cryopreserved for >2, 3, or 5 years (Supplementary Fig. S2c, d, and f–j).

LBR was significantly lower among embryos cryopreserved for >3 months compared with those cryostored for ≤3 months (OR = 0.90 95% CI: 0.84–0.95, *P* = 0.0008; *I*^2^ = 84%, *P* < 0.0001) (Fig. 2f). This decrement persisted when the cryopreservation duration threshold was advanced to >6 months (OR = 0.74, 95% CI: 0.69–0.80, *P* < 0.00001; *I*^2^ = 70%, *P* = 0.0005), >12 months (OR = 0.72, 95% CI: 0.63–0.84, *P* < 0.0001; *I*^2^ = 83%, *P* < 0.0001), or >2 years (OR = 0.80, 95% CI: 0.72–0.89, *P* < 0.0001; *I*^2^ = 0%, *P* = 0.52) (Fig. 2g–i). Irrespective of whether the cut-off was set at 3 months (OR = 0.90 95% CI: 0.84–0.95, *P* = 0.0008; *I*^2^ = 84%, *P* < 0.0001), 6 months (OR = 0.83, 95% CI: 0.74–0.93, *P* = 0.001; *I*^2^ = 88%, *P* < 0.0001), or 12 months (OR = 0.79, 95% CI: 0.72–0.87, *P* < 0.00001; *I*^2^ = 76%, *P* < 0.0001), prolonged cryopreservation remained associated with a reduced likelihood of live birth (Fig. 2f and Supplementary Fig. S3a and e). Consistent with comparison of the clinical pregnancy, LBR was comparable between embryos cryopreserved for ≤6 months (or any longer durations) and those cryopreserved for >2, 3, or 5 years (Supplementary Fig. S3d and g–k).

We also performed analyses on BPR, embryo IR, EPR, and MR. The pattern of differences in BPR resembled that observed for CPR. Specifically, embryos cryopreserved for >3 months exhibited a significantly lower biochemical pregnancy rate compared to those cryopreserved for ≤3 months (OR = 0.82, 95% CI: 0.73–0.92, *P* = 0.0007; *I*^2^ = 92%, *P* < 0.0001) (Supplementary Fig. S4a). This trend was consistently observed across longer cryopreservation durations, including >6 months (OR = 0.70, 95% CI: 0.63–0.77, *P* < 0.00001; *I*² = 77%, *P* = 0.0002), >12 months (OR = 0.72, 95% CI: 0.65–0.80, *P* < 0.00001; *I*² = 63%, *P* = 0.01), and >2 years (OR = 0.74, 95% CI: 0.61–0.90, *P* = 0.002; *I* = 64%, *P* = 0.04) (Supplementary Fig. S4b–d). Furthermore, regardless of whether the stratification threshold was set at 3 months (OR = 0.82, 95% CI: 0.73–0.92, *P* = 0.0007; *I*² = 92%, *P* < 0.0001), 6 months (OR = 0.74, 95% CI: 0.67–0.82, *P* < 0.00001; *I*² = 79%, *P* < 0.0001), or 12 months (OR = 0.77, 95% CI: 0.70–0.83, *P* < 0.00001; *I*² = 73%, *P* = 0.0006), longer cryopreservation duration was consistently associated with a significantly lower BPR (Supplementary Fig. S4a, e, and h). However, BPR was comparable between embryos cryopreserved for >2 years and those cryopreserved for ≤6 months (or longer durations) (Supplementary Fig. S4g, i, and j). We also found that, compared to embryos cryopreserved for ≤3 months, those stored for >3 months (OR = 0.69, 95% CI: 0.67–0.72, *P* < 0.00001; *I*² = 43%, *P* = 0.17) and those stored for >6 months (OR = 0.71, 95% CI: 0.54–0.94, *P* = 0.02; *I*² = 74%, *P* = 0.02) had significantly lower IR (Supplementary Fig. S5a and b). In contrast, embryo IR was comparable across other cryopreservation duration comparisons (Supplementary Fig. S5c–n). Across all storage duration groups evaluated in this study, the incidence of ectopic pregnancy was comparable among the different groups (Supplementary Fig. S6). Regarding MR, embryos cryopreserved for >12 months had a higher MR compared to those stored for ≤12 months (OR = 1.16, 95% CI: 1.09–1.25, *P* < 0.0001; *I*² = 0%, *P* = 0.62) (Supplementary Fig. S7g). However, this pattern was not observed in any of the other cryopreservation duration comparisons (Supplementary Fig. S7a–f and h–l).

#### The comparison of neonatal outcomes

There were 15 studies reporting neonatal outcomes (Wirleitner *et al.*, 2013; Liu *et al.*, 2014; Li *et al.*, 2017, 2020, 2023; Ueno *et al.*, 2018; Cui *et al.*, 2021; Lee *et al.*, 2021; Mao *et al.*, 2022; Shi *et al.*, 2022; Xu *et al.*, 2022; Ma *et al.*, 2023; Zhan *et al.*, 2024; Wang *et al.*, 2024b; Guo *et al.*, 2025). Because plurality directly influences the risks of preterm delivery, birth weight extremes, and other neonatal measures, we performed subgroup and pooled analyses stratified by outcomes from singletons only, multiples only, or both.

When the ‘both’ subgroup was analyzed, preterm-birth rate was higher in embryos cryopreserved for >12 months (OR = 1.10, 95% CI: 1.00–1.20, *P* = 0.04; *I*^2^ = 41%, *P* = 0.16) than in those cryopreserved for ≤12 months (Supplementary Fig. S8g). In the pooled analyses, embryos cryopreserved for >12 months also showed a higher preterm-birth rates relative to those cryopreserved for ≤12 months (OR = 1.09, 95% CI: 1.00–1.19, *P* = 0.04; *I*^2^ = 30%, *P* = 0.19) (Supplementary Fig. S8g). Irrespective of whether subgroup and pooled analyses were examined, no significant differences in preterm-birth rates were detected when embryos cryopreserved for ≤3, ≤6 or ≤2 years were compared with their longer-storage counterparts (Supplementary Fig. S8). Similarly, HBWR and CMR did not differ between any of the predefined cryopreservation intervals (shown in Supplementary Figs S9 and S10).

When the ‘both’ subgroup was analyzed, LBWR was lower in embryos cryopreserved for >6 months (OR = 0.83, 95% CI: 0.70–0.99, *P* = 0.04) and >12 months (OR = 0.75, 95% CI: 0.57–0.98, *P* = 0.03) than in those cryopreserved for ≤3 months (Supplementary Fig. S11b and c). In the pooled analyses, embryos cryopreserved for >2 years also showed a lower LBWR relative to those cryopreserved for ≤12 months (OR = 0.74, 95% CI: 0.54–1.00, *P* = 0.05; *I*^2^ = 0%, *P* = 0.72) (Supplementary Fig. S11i). All comparisons between other cryopreservation durations, within either subgroup or pooled analyses, revealed comparable LBWR (Supplementary Fig. S11).

Subgroup meta-analysis identified a modest but significant reduction in the proportion of male infants among embryos cryopreserved for >3 months versus ≤3 months (OR = 0.92, 95% CI: 0.86–0.98, *P* = 0.02; *I*^2^ = 0%, *P* = 0.95) in the ‘both’ group (Supplementary Fig. S12a). This pattern was consistently observed when embryos cryopreserved for >2 years were compared with those cryopreserved for ≤3 months (OR = 0.79, 95% CI: 0.64–0.97, *P* = 0.02; *I*^2^ = 0%, *P* = 0.56), ≤6 months (OR = 0.82, 95% CI: 0.68–0.98, *P* = 0.03; *I*^2^ = 0%, *P* = 0.78), ≤12 months (OR = 0.81, 95% CI: 0.67–0.98, *P* = 0.03; *I*^2^ = 0%, *P* = 0.80), and ≤2 years (OR = 0.81, 95% CI: 0.68–0.98, *P* = 0.03; *I*^2^ = 0%, *P* = 0.76) (Supplementary Fig. S12d, g, i, and j). Across all remaining pairwise contrasts, both within individual subgroups and in the overall pooled analyses, the proportion of male newborns did not differ significantly between cryopreservation duration groups (Supplementary Fig. S12).

### Publication bias

For outcomes that included more than 10 studies (clinical pregnancy rate, live birth rate, and miscarriage rate), we created funnel plots (Supplementary Fig. S13) and did not find distinct publication bias.

### Sensitivity analysis

Sensitivity analysis assessed embryo survival, clinical pregnancy, live birth, biochemical pregnancy, embryo implantation, congenital malformation, and male child rates (Supplementary Table S2). The sensitivity assessment found the results to be stable.

## Discussion

In the current meta-analysis, we evaluated whether prolonged cryopreservation compromises embryo survival, pregnancy outcomes, and neonatal parameters relative to shorter cryopreservation duration. Collectively, embryo survival and main pregnancy outcomes differed markedly across cryopreservation durations, with a consistent trend toward poorer performance with increasing cryopreservation time. Specifically, post-thaw survival declined significantly when embryos had been cryopreserved for more than 5 years. Compared with embryos cryopreserved for less than 3 months (or any shorter reference duration), those with longer cryopreservation exhibited lower CPR, LBR, BPR, and embryo IR, whereas the MR tended to increase. Among neonatal outcomes, extended cryopreservation was associated with an increased risk of preterm birth, a reduced risk of low birth weight, and a modestly lower male sex ratio. By contrast, HBWR and CMR remained unaffected. Collectively, these findings suggest that the impact of cryopreservation duration on pregnancy outcomes may follow a non-linear ‘threshold’ or ‘plateau’ effect. Adverse influences are most evident during the initial cryopreservation period (e.g. within 3–12 months), whereas prolongation beyond this window (e.g. >2 years) does not confer additional deterioration in pregnancy outcomes.

The present meta-analysis demonstrated a statistically significant decline in post-thaw survival when embryos were cryopreserved for more than 5 years, a finding that aligns with an emerging body of both clinical and pre-clinical evidence. Mao *et al.* (2022) observed a progressive decrement in survival and clinical pregnancy once storage exceeded 24 months, and data from Shi *et al.* and propensity-matched data from Cui *et al.* identified a clear inflection point after 60 months (Cui *et al.*, 2021; Shi *et al.*, 2022). Similar trends have been reported in murine models, where prolongation of storage proportionally reduced blastocyst re-expansion and increased chromosomal aberration (Mozdarani and Moradi, 2007; Zarei Moradi *et al.*, 2013). Although metabolism is effectively arrested in liquid nitrogen (LN_2_), the physical and chemical stresses inherent to vitrification may nevertheless accumulate, including cumulative low-dose background radiation (≈0.1 rad/year) (Quintans *et al.*, 2002) and time-dependent lipid/protein drift within the cryogenic milieu. High concentrations of permeating cryoprotectants (CPAs), most commonly dimethyl sulfoxide (DMSO) and ethylene glycol (EG), are required to achieve glass transition, yet DMSO in particular is known to trigger mitophagy, autophagy and measurable shifts in DNA methylation (Iwatani *et al.*, 2006; Kang *et al.*, 2017, 2020; Verheijen *et al.*, 2019). The balance between dehydration and ice formation is critical: excessive cellular dehydration raises intra-cellular CPA concentrations to potentially cytotoxic levels (Lane *et al.*, 1999; Liebermann *et al.*, 2002; Shaw and Jones, 2003), whereas insufficient dehydration allows lethal ice nucleation. Over intervals measured in years, even nano-scale ice re-crystallization or CPA seepage along micro-fractures in the glassy matrix may gradually erode viability. Incomplete elution of these CPAs after warming can generate an osmotic shock that compromises membrane integrity and reduces developmental competence (Jenkins *et al.*, 2012; Yong *et al.*, 2017). Equally, repeated opening of storage tanks for inventory or LN₂ replenishment subjects straws to transient temperature oscillations that may permit devitrification or intracellular ice micro-crystal growth, both of which are injurious to plasma membranes and cytoskeletal structures (Gosden, 2011).

Systematic analysis revealed that, compared with embryos cryopreserved for <3 months, progressively longer cryopreservation intervals (>6 months, >12 months and >2 years) were associated with lower CPR, LBR, BPR and embryo IR; storage beyond 12 months was also linked to a higher MR. However, once the cryopreservation period reached 2, 3 or even 5 years, differences in CPR, LBR and BPR were no longer significant when compared with embryos stored for ≤ 6 months (or any shorter reference). These observations are consistent with several contemporary reports. Two large cohorts (Li *et al.*, 2020; Zhang *et al.*, 2021) similarly described an inverse relationship between storage duration and BPR, CLR and LBR. Li *et al.* (2020) specifically noted that impairment becomes detectable after only 3 months, a finding that closely matches our core result. An additional study has shown that prolonged vitrification in ‘freeze-all’ cycles is associated with reduced CPR and LBR (Hu *et al.*, 2022). Collectively, these data reinforce the concept that cryopreservation time is an independent determinant of reproductive success. Nevertheless, the literature is not uniform. A previous meta-analysis concluded that storage length has no material influence on embryo viability or pregnancy outcome (Ma *et al.*, 2021). In addition to the meta-analysis by Ma *et al.*, a more recent systematic review and meta-analysis reported that prolonged cryo-storage (>12 months) did not significantly affect cryo-survival, miscarriage, live birth, or major malformation rates, however, in the subgroup analysis restricted to untested vitrified blastocysts, a difference in survival rate was observed, whereas clinical outcome rates remained unaffected (Canosa *et al.*, 2022). Other investigators reported adverse effects only when storage exceeded 6 years (Li *et al.*, 2020; Cui *et al.*, 2021). Our study extends these observations by providing a more detailed time‑dependent analysis across multiple thresholds (3 months, 6 months, 12 months, 2 years, 3 years and 5 years), offering a more granular perspective that may help reconcile some of the apparent discrepancies in the literature.

Such discrepancies probably reflect heterogeneity in study design, patient characteristics (age, aetiology of infertility), cryopreservation technique (slow freezing vs. vitrification), choice of cut-off points, and the extent of statistical adjustment, especially for maternal age. Our stratified comparisons across multiple pre-specified time-points yielded high inter-study heterogeneity, underscoring these differences. The underlying mechanisms remain speculative. One plausible hypothesis is that cryopreservation inflicts cumulative molecular or sub-cellular damage that manifests principally during the first year, thereby explaining the early decline in pregnancy and live-birth rates and the parallel increase in miscarriage observed here and elsewhere (Li *et al.*, 2020; Cui *et al.*, 2021; Seshadri *et al.*, 2021). Beyond this initial window, embryos that survive warming and remain transferable may represent a selected population with superior stress tolerance, creating a ‘survivor bias’ that stabilizes outcomes in the ultra-long storage group (Zhan *et al.*, 2024). It is important to distinguish the embryo-centric survivor bias described above from patient-centric confounding related to maternal age and transfer timing. While longer storage frequently correlates with older age at transfer, the relationship is not unidirectional: older women may choose to proceed with transfer sooner due to age-related reproductive urgency, whereas younger women with good prognosis may intentionally delay frozen embryo transfer after achieving a live birth from a fresh cycle. These patient-driven decisions represent a distinct source of confounding that operates alongside the biological selection of cryotolerant embryos. Both mechanisms may jointly contribute to the observed patterns, and our statistical adjustment for chronological age can only partially account for these complex interactions. Besides, longer storage is temporally correlated with older maternal age at transfer, and advanced age at the time of transfer is well known to compromise endometrial receptivity (Seshadri *et al.*, 2021). It is important to note that this represents an additional confounding factor that complicates the interpretation of storage‑duration effects. Moreover, reliable clinical tests for endometrial receptivity (e.g. the endometrial receptivity analysis (ERA) test) remain controversial, and effective therapies for poor receptivity are lacking (Díaz-Gimeno *et al.*, 2017; Simón *et al.*, 2020), making this confounder difficult to adjust for in practice. Although our models attempted to adjust for chronological age, the strong collinearity between age and storage duration may still partly explain the observed associations. However, Liang *et al.* (2024) recently demonstrated that advancing maternal age and differential blastocyst transfer rates do not fully account for the reduced success seen after 6 months of storage, suggesting that storage duration exerts an independent, albeit modest, biological effect.

In the present analysis, prolonged cryopreservation was not associated with significant alterations in rates of high birth weight or congenital malformations, a result that accords with several previous reports (Ueno *et al.*, 2018; Li *et al.*, 2020). Three associations nevertheless merit emphasis. First, in both sub-analysis and pool-analysis, cryopreservation exceeding 12 months was associated with a slightly increased risk of preterm birth. Second, in selected sub-analyses, embryos stored for >6 or >12months yielded newborns with a lower LBWR than those cryopreserved for shorter intervals. Third, cryopreservation beyond 2 years was linked to a measurable reduction in the proportion of male live births. The inverse relationship between low birth weight and cryopreservation duration appears discrepant with studies suggesting that longer cryopreservation may increase the risk of large-for-gestational-age (LGA) neonates (Wei *et al.*, 2019; Hu *et al.*, 2020; Zaat *et al.*, 2021), yet it underscores the complexity of the phenomenon. A plausible explanation is healthy-embryo selection bias. In clinical practice, embryos judged to be of the highest morphological quality are often transferred first, whereas those set aside for long-term banking, although meeting cryopreservation criteria, may harbour greater heterogeneity in developmental competence. Over extended cryopreservation, embryos more susceptible to cryoinjury are progressively lost, either failing to survive warming or succumbing in early gestation, thereby enriching the cohort that reaches term with infants of average, or even higher, birth weight. Additional confounders include disparate freezing protocols (slow-cooling vs. vitrification), endometrial preparation regimens, and maternal characteristics such as age and body mass index. Collectively, our data indicate that cryopreservation duration is unlikely to be an independent and robust predictor of birth weight.

The most striking observation was the consistent decline in male sex ratio with increasing cryopreservation time, a finding that achieved statistical significance across multiple cut-off points. The mechanism remains speculative; two complementary mechanisms may explain these findings. First, if male embryos indeed have a tendency to develop faster and achieve higher morphological scores, as suggested by Alfarawati *et al.* (2011), this might make them more likely to be prioritized for earlier transfer (Spangmose *et al.*, 2020), thereby leaving a higher proportion of female embryos in prolonged storage. Second, male conceptuses may be more vulnerable to the non-physiological stress of extended cryopreservation. This hypothesis is supported by the well-documented observation that male fetuses experience higher rates of pregnancy loss at all gestational stages, including elevated risks of both miscarriage (early gestation) and stillbirth (late gestation), compared with female fetuses (Mondal *et al.*, 2014; Sánchez Barricarte, 2026). This sex-specific vulnerability is thought to reflect inherent biological differences in placental development and stress tolerance, which may render male embryos less resilient to the cumulative stresses of prolonged cryopreservation. Consequently, the non-physiological stress of prolonged cryopreservation may disproportionately impair their viability, skewing the sex ratio among survivors. Although this hypothesis requires prospective validation, it provides a novel perspective on how laboratory manipulation of gametes and embryos might influence demographic parameters at birth.

Importantly, the absence of a detectable association between storage duration and major congenital anomalies does not preclude subtler, long-term health effects. Cryopreservation can perturb the epigenetic landscape, such as altering DNA methylation and histone modifications. Multi-omic studies have shown that such deviations are enriched in pathways governing neural, cardiovascular and metabolic function (Wang *et al.*, 2010; Maldonado *et al.*, 2015; Hiura *et al.*, 2017; Chen *et al.*, 2020). While these changes are not manifest as structural malformations at delivery, they could predispose ART offspring to later-onset disorders such as obesity, hypertension, or neurodevelopmental delay. Reports of increased prevalence of rare imprinting disorders (e.g. Beckwith–Wiedemann and Angelman syndromes) in ART-conceived children further emphasize the need for extended follow-up to uncover latent sequelae of embryo cryopreservation (Hiura *et al.*, 2012; Hattori *et al.*, 2019).

### Strengths and limitations

To the best of our knowledge, this is the most comprehensive systematic review and meta-analysis to date examining the full spectrum of embryonic cryopreservation duration on assisted reproductive outcomes and neonatal health, covering the entire chain from embryo warming, pregnancy establishment, live birth, and neonatal indices. Second, the inclusion of 254 017 frozen-thawed embryo transfer cycles provides a large sample size, enhancing statistical power. Finally, we employed multiple pre-specified time cut-offs for stratified comparisons and conducted singleton/multiple-birth subgroup analyses for neonatal outcomes, which helped to uncover context-specific association patterns, most notably a ‘plateau effect’ whereby outcomes stabilized with ultra-long storage (>2 years) and a novel, consistent association of declining male sex ratio. This study also has certain limitations. The primary limitation is the substantial heterogeneity among included studies. The substantial heterogeneity observed may be attributed to variations in embryo grading criteria, cryopreservation/thawing protocols, and patient selection across studies. However, incomplete reporting of these parameters limited our ability to perform further subgroup analyses. Although random-effects models were applied to account for between-study variance, residual confounding from unmeasured factors remains. Additionally, several potential confounders could not be systematically evaluated due to insufficient reporting: FET cycle type (natural vs. hormone replacement therapy) was not stratified by regimen in most studies, maternal age was reported heterogeneously (e.g. mean ± SD, median with interquartile range (IQR), or age groups), with the timing of measurement (oocyte retrieval vs. FET) often unspecified, and embryo quality at freezing, thawing, or transfer was not consistently reported or stratified across studies. Second, all included studies were of retrospective observational design; thus, causality cannot be established, and residual confounding, especially maternal age, which is highly collinear with storage duration, may have influenced the observed associations. Finally, for analyses of neonatal outcomes (particularly male sex ratio), some studies combined singleton and multiple-birth data; although we conducted subgroup analyses, caution is warranted when interpreting these results.

## Conclusion

In summary, this meta-analysis demonstrates a non-linear relationship between embryo cryopreservation duration and multiple pregnancy outcomes. During the early cryopreservation phase (especially within 3–12 months), longer storage is associated with lower embryo survival, clinical pregnancy, live birth, and biochemical pregnancy rates, along with a potentially increased miscarriage rate. However, once storage duration extends to 2 years or more, these outcomes do not worsen further, suggesting the existence of a ‘plateau phase’. Regarding neonatal outcomes, prolonged cryopreservation does not increase the risks of preterm birth, high birth weight, or congenital malformations, but is significantly associated with a decreased male sex ratio at birth, a phenomenon whose mechanism remains to be elucidated. These findings have important clinical implications: when feasible, patients should be encouraged to undergo transfer as early as possible after embryo cryopreservation to optimize pregnancy outcomes. Meanwhile, for couples who need to store embryos for extended periods due to medical or personal reasons, this study provides reassuring evidence that ultra-long cryopreservation does not lead to further deterioration in pregnancy and neonatal outcomes.

## Supplementary Material

hoag077_Supplementary_Data

## Data Availability

All data generated or analyzed during this study are included in this published article and its supplementary information files.
