# Supplementary material for: Effect of cryopreservation duration on pregnancy outcomes in embryos: a systematic review and meta-analysis
Source: Hum Reprod Open. 2026 Aug 13;2026(4):hoag077. doi: 10.1093/hropen/hoag077 (PMC13588701; doi:10.1093/hropen/hoag077)
Supplement: Supplementary file 1 [file hoag077_supplementary_data.zip › Supplementary Figure S6.pdf]

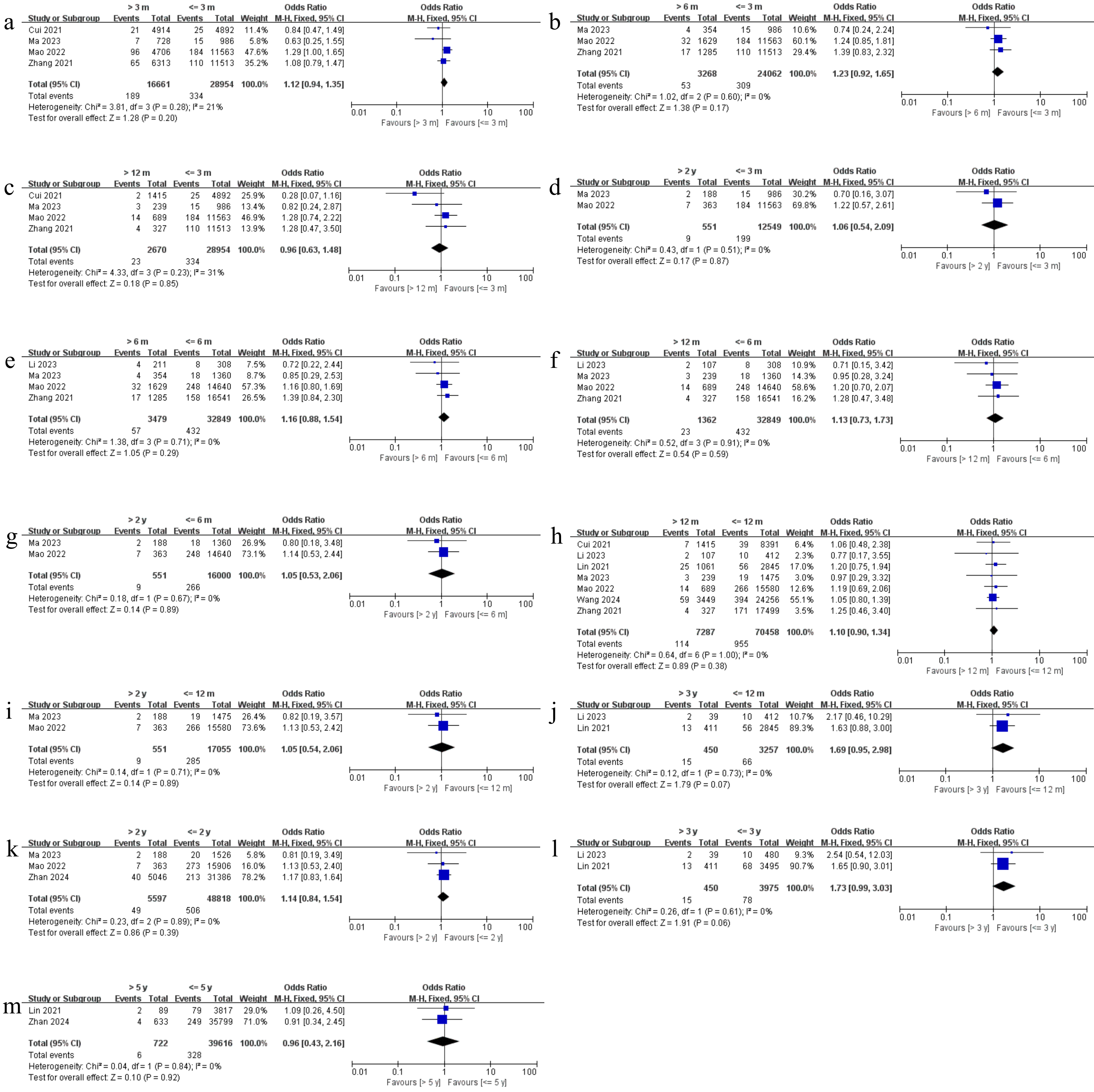

Supplementary Figure S6. Ectopic pregnancy comparison between embryos cryopreserved for different duration by pooled analysis. a) > 3 m v.s. <= 3 m, b) > 6 m v.s. <= 3 m, c) > 12 m v.s. <= 3 m, d) > 2 y v.s. <= 3 m, e) > 6 m v.s. <= 6 m, f) > 12 m v.s. <= 6 m, g) > 2 y v.s. <= 6 m, h) > 12 m v.s. <= 12 m, i) > 2 y v.s. <= 12 m, j) > 3 y v.s. <= 12 m, k) > 2 y v.s. <= 2 y, l) > 3 y v.s. <= 3 y, m)> 5 y v.s. <= 5 y.
