# Supplementary material for: Effect of cryopreservation duration on pregnancy outcomes in embryos: a systematic review and meta-analysis
Source: Hum Reprod Open. 2026 Aug 13;2026(4):hoag077. doi: 10.1093/hropen/hoag077 (PMC13588701; doi:10.1093/hropen/hoag077)
Supplement: Supplementary file 1 [file hoag077_supplementary_data.zip › Supplementary Figure S13.pdf]

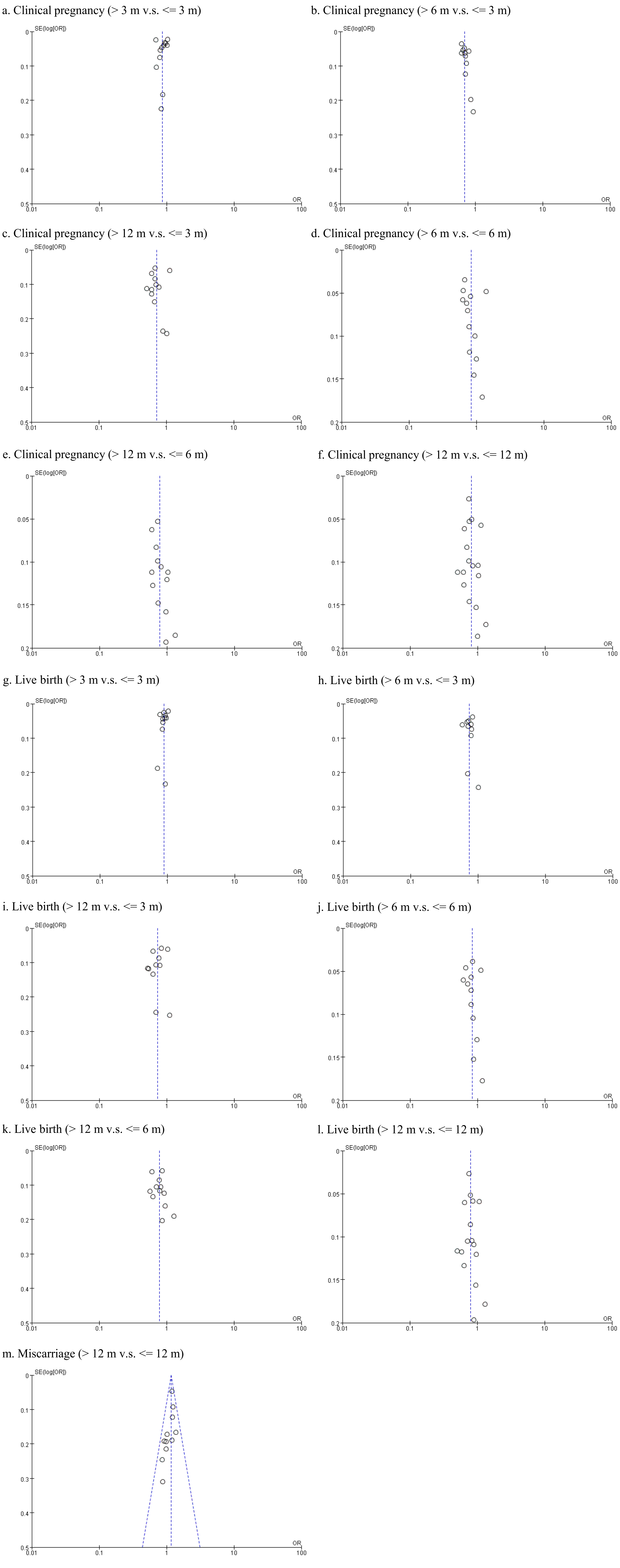

Supplementary Figure S13. Funnel plot of clinical pregnancy, live birth and miscarriage comparison. a) studies of clinical pregnancy comparison (> 3 m v.s. <= 3 m), b) studies of clinical pregnancy comparison (> 6 m v.s. <= 3 m), c) studies of clinical pregnancy comparison (> 12 m v.s. <= 3 m), d) studies of clinical pregnancy comparison (> 6 m v.s. <= 6 m), e) studies of clinical pregnancy comparison (> 12 m v.s. <= 6 m), f) studies of clinical pregnancy comparison (> 12 m v.s. <= 12 m), g) studies of live birth comparison (> 3 m v.s. <= 3 m), h) studies of live birth comparison (> 6 m v.s. <= 3 m), i) studies of live birth comparison (> 12 m v.s. <= 3 m), j) studies of live birth comparison (> 6 m v.s. <= 6 m), k) studies of live birth comparison (> 12 m v.s. <= 6 m), l) studies of live birth comparison (> 12 m v.s. <= 12 m), m) studies of miscarriage comparison (> 12 m v.s. <= 12 m).
