# Supplementary material for: Effect of cryopreservation duration on pregnancy outcomes in embryos: a systematic review and meta-analysis
Source: Hum Reprod Open. 2026 Aug 13;2026(4):hoag077. doi: 10.1093/hropen/hoag077 (PMC13588701; doi:10.1093/hropen/hoag077)
Supplement: Supplementary file 1 [file hoag077_supplementary_data.zip › Supplementary-Tables-post_adjudication_clean_ EO.docx]

**Supplementary Table S1. Detailed search strategy**

| **Step** | **Search detail** |
| --- | --- |
| 1 | #1: Mesh: Embryonic Structures  #2: (Embryonic Structure) OR (Structure, Embryonic) OR Embryo OR Embryos OR (Prenatal Structures) OR (Prenatal Structure) OR (Structure, Prenatal) OR (Presumptive Structures) OR (Presumptive Structure) OR (Structure, Presumptive)  #3: #1 OR #2  #4: Mesh: Blastocyst  #5: Blastocysts OR (Embryo, Preimplantation) OR (Embryos, Preimplantation) OR (Preimplantation Embryo) OR (Preimplantation Embryos)  #6: #4 OR #5  #7 : #3 OR #6 |
| 2 | #8:Mesh: vitrification  #9: (Glass Transition) OR (Transition, Glass) OR (Glass-Liquid Transition) OR (Glass Liquid Transition) OR (Transition, Glass-Liquid) OR (Liquid-Glass Transition) OR (Liquid Glass Transition) OR (Transition, Liquid-Glass)  #10: #8 OR #9  #11:Mesh: cryopreservation  #12: Cryofixation OR (Cryonic Suspension) OR (Cryonic Suspensions) OR (Suspension, Cryonic)  #13: #11 OR #12  #14: Mesh: Freezing  #15: Melting  #16: #14 OR #15  #17: #10 OR #13 OR #16 |
| 3 | #18: Mesh: pregnancy  #19: Pregnancies OR Gestation  #20: #18 OR #19  #21: Mesh: live birth  #22: Live Births  #23: #21 OR #22  #24: #20 OR #23 |
| 4 | #7 AND #17 AND #24 |

**Supplementary Table S2.** **Sensitivity analysis of the meta-analysis results**

| **Study omitted** | ***OR^[1]^*** | ***95%CI*** | ***P value*** | ***I^2^*** |  |
| --- | --- | --- | --- | --- | --- |
| **embryo survival rate (3m^[2]^)** | | | | |  |
| Cui et al., 2021 | 0.65 | 0.16, 2.67 | < 0.00001 | 99% |  |
| Li et al., 2016 | 0.65 | 0.16, 2.59 | < 0.00001 | 99% |  |
| Li et al., 2020 | 0.59 | 0.15, 2.31 | < 0.00001 | 99% |  |
| Ma et al., 2023 | 0.63 | 0.16, 2.49 | < 0.00001 | 99% |  |
| Mao et al., 2022 | 1.12 | 0.94, 1.33 | 0.51 | 0% |  |
| Wirleitner et al., 2013 | 0.59 | 0.15, 2.40 | < 0.00001 | 99% |  |
| **Random effects model^[3]^** | **0.67** | **0.19, 2.35** | **< 0.00001** | **99%** |  |
| **embryo survival rate (3m vs 6m)** | | | | | |
| Li et al., 2016 | 0.53 | 0.07, 4.03 | < 0.00001 | 99% |  |
| Li et al., 2020 | 0.49 | 0.07, 3.60 | < 0.00001 | 99% |  |
| Ma et al., 2023 | 0.52 | 0.07, 3.93 | < 0.00001 | 99% |  |
| Mao et al., 2022 | 1.11 | 0.88, 1.40 | 0.81 | 0% |  |
| Wirleitner et al., 2013 | 0.52 | 0.06, 4.40 | < 0.00001 | 99% |  |
| **Random effects model** | **0.60** | **0.10, 3.64** | **< 0.00001** | **99%** |  |
| **embryo survival rate (3m vs 2y^[4]^)** | | | | | |
| Ma et al., 2023 | 0.19 | 0.00, 7.50 | < 0.00001 | 100% |  |
| Mao et al., 2022 | 1.02 | 0.72, 1.46 | 0.97 | 0% |  |
| Wirleitner et al., 2013 | 0.18 | 0.01, 5.68 | < 0.00001 | 96% |  |
| **Random effects model** | **0.32** | **0.02, 6.72** | **< 0.00001** | **99%** |  |
| **embryo survival rate (6m)** | | | | | |
| Lee et al., 2021 | 0.67 | 0.17, 2.62 | < 0.00001 | 99% |  |
| Li et al., 2016 | 0.62 | 0.16, 2.31 | < 0.00001 | 99% |  |
| Li et al., 2020 | 0.63 | 0.17, 2.36 | < 0.00001 | 99% |  |
| Ma et al., 2023 | 0.65 | 0.17, 2.44 | < 0.00001 | 99% |  |
| Mao et al., 2022 | 0.97 | 0.76, 1.24 | 0.10 | 48% |  |
| Wirleitner et al., 2013 | 0.70 | 0.17, 3.00 | < 0.00001 | 99% |  |
| **Random effects model** | **0.71** | **0.21, 2.39** | **< 0.00001** | **99%** |  |
| **embryo survival rate (6m vs 12m)** | | | | | |
| Lee et al., 2021 | 0.55 | 0.12, 2.46 | < 0.00001 | 99% |  |
| Li et al., 2016 | 0.48 | 0.11, 2.07 | < 0.00001 | 99% |  |
| Li et al., 2020 | 0.60 | 0.13, 2.79 | < 0.00001 | 99% |  |
| Ma et al., 2023 | 0.54 | 0.12, 2.38 | < 0.00001 | 99% |  |
| Mao et al., 2022 | 0.82 | 0.62, 1.08 | 0.17 | 37% |  |
| Wirleitner et al., 2013 | 0.58 | 0.12, 2.86 | < 0.00001 | 99% |  |
| **Random effects model** | **0.60** | **0.15, 2.34** | **< 0.00001** | **99%** |  |
| **embryo survival rate (6m vs 2y)** | | | | | |
| Lee et al., 2021 | 0.35 | 0.04, 3.21 | < 0.00001 | 99% |  |
| Ma et al., 2023 | 0.34 | 0.04, 2.92 | < 0.00001 | 100% |  |
| Mao et al., 2022 | 0.79 | 0.62, 1.00 | 0.69 | 0% |  |
| Wirleitner et al., 2013 | 0.38 | 0.04, 3.86 | < 0.00001 | 99% |  |
| **Random effects model** | **0.44** | **0.07, 2.96** | **< 0.00001** | **99%** |  |
| **embryo survival rate (12m)** | | | | | |
| Cui et al., 2021 | 0.72 | 0.27, 1.87 | < 0.00001 | 99% |  |
| Lee et al., 2021 | 0.70 | 0.26, 1.88 | < 0.00001 | 99% |  |
| Li et al., 2016 | 0.65 | 0.25, 1.68 | < 0.00001 | 99% |  |
| Li et al., 2020 | 0.75 | 0.29, 1.96 | < 0.00001 | 99% |  |
| Lin et al., 2021 | 0.68 | 0.26, 1.78 | < 0.00001 | 99% |  |
| Ma et al., 2023 | 0.70 | 0.27, 1.80 | < 0.00001 | 99% |  |
| Mao et al., 2022 | 0.91 | 0.78, 1.07 | 0.11 | 41% |  |
| Shi et al., 2022 | 0.70 | 0.25, 1.93 | < 0.00001 | 99% |  |
| Wirleitner et al., 2013 | 0.72 | 0.26, 1.96 | < 0.00001 | 99% |  |
| **Random effects model** | **0.72** | **0.29, 1.77** | **< 0.00001** | **99%** |  |
| **embryo survival rate (12m vs 2y)** | | | | | |
| Lee et al., 2021 | 0.41 | 0.06, 2.64 | < 0.00001 | 99% |  |
| Ma et al., 2023 | 0.39 | 0.06, 2.41 | < 0.00001 | 99% |  |
| Mao et al., 2022 | 0.82 | 0.65, 1.02 | 0.83 | 0% |  |
| Wirleitner et al., 2013 | 0.42 | 0.06, 2.92 | < 0.00001 | 99% |  |
| **Random effects model** | 0.49 | 0.10, 2.44 | **< 0.00001** | **99%** |  |
| **embryo survival rate (12m vs 3y)** | | | | | |
| Lin et al., 2021 | 0.89 | 0.77, 1.04 | 0.84 | 0% |  |
| Shi et al., 2022 | 1.23 | 0.72, 2.11 | 0.08 | 67% |  |
| Wirleitner et al., 2013 | 1.16 | 0.65, 2.07 | 0.010 | 85% |  |
| **Random effects model** | **1.07** | **0.75, 1.53** | **0.04** | **70%** |  |
| **embryo survival rate (2y)** | | | | | |
| Lee et al., 2021 | 0.62 | 0.17, 2.23 | < 0.00001 | 100% |  |
| Liu et al., 2014 | 0.59 | 0.16, 2.21 | < 0.00001 | 99% |  |
| Ma et al., 2023 | 0.60 | 0.18, 2.06 | < 0.00001 | 100% |  |
| Mao et al., 2022 | 0.99 | 0.89, 1.09 | 0.42 | 0% |  |
| Wirleitner et al., 2013 | 0.62 | 0.17, 2.31 | < 0.00001 | 100% |  |
| Zhan et al., et al., 2024 | 0.61 | 0.15, 2.43 | < 0.00001 | 99% |  |
| **Random effects model** | **0.66** | **0.21, 2.05** | **< 0.00001** | **99%** |  |
| **embryo survival rate (3y)** | | | | | |
| Lin et al., 2021 | 0.94 | 0.84, 1.04 | 0.55 | 0% |  |
| Liu et al., 2014 | 1.10 | 0.77, 1.58 | 0.03 | 72% |  |
| Shi et al., 2022 | 1.14 | 0.86, 1.52 | 0.12 | 53% |  |
| Wirleitner et al., 2013 | 1.04 | 0.82, 1.32 | 0.03 | 71% |  |
| **Random effects model** | **1.03** | **0.85, 1.25** | **0.07** | **58%** |  |
| **clinical pregnancy (3 m)** | | | | | |
| Costa et al., 2025 | 0.88 | 0.79, 0.98 | < 0.00001 | 94% |  |
| Cui et al., 2021 | 0.85 | 0.76, 0.95 | < 0.00001 | 94% |  |
| Guo et al., 2025 | 0.87 | 0.78, 0.97 | < 0.00001 | 94% |  |
| Hu et al., 2022 | 0.86 | 0.77, 0.96 | < 0.00001 | 94% |  |
| Li et al., 2016 | 0.86 | 0.78, 0.96 | < 0.00001 | 94% |  |
| Liang et al., 2024 | 0.86 | 0.77, 0.96 | < 0.00001 | 94% |  |
| Ma et al., 2023 | 0.87 | 0.78, 0.97 | < 0.00001 | 94% |  |
| Mao et al., 2022 | 0.90 | 0.84, 0.96 | < 0.00001 | 79% |  |
| Wirleitner et al., 2013 | 0.87 | 0.78, 0.96 | < 0.00001 | 94% |  |
| Zhang et al., 2021 | 0.85 | 0.77, 0.96 | < 0.00001 | 94% |  |
| Zhao et al., 2026 | 0.85 | 0.77, 0.94 | < 0.00001 | 92% |  |
| Zheng et al., 2023 | 0.87 | 0.78, 0.97 | < 0.00001 | 94% |  |
| **Random effects model** | **0.87** | **0.78, 0.96** | **< 0.00001** | **94%** |  |
| **clinical pregnancy (3 m vs 6 m)** | | | | | |
| Costa et al., 2025 | 0.70 | 0.65, 0.74 | 0.01 | 57% |  |
| Guo et al., 2025 | 0.70 | 0.66, 0.75 | 0.01 | 57% |  |
| Hu et al., 2022 | 0.70 | 0.65, 0.75 | 0.01 | 56% |  |
| Li et al., 2016 | 0.69 | 0.65, 0.74 | 0.02 | 55% |  |
| Liang et al., 2024 | 0.68 | 0.64, 0.71 | 0.18 | 28% |  |
| Ma et al., 2023 | 0.69 | 0.65, 0.74 | 0.02 | 55% |  |
| Mao et al., 2022 | 0.71 | 0.67, 0.75 | 0.18 | 29% |  |
| Wirleitner et al., 2013 | 0.69 | 0.65, 0.74 | 0.02 | 53% |  |
| Zhang et al., 2021 | 0.71 | 0.66, 0.75 | 0.02 | 53% |  |
| Zhao et al., 2026 | 0.70 | 0.65, 0.75 | 0.01 | 57% |  |
| Zheng et al., 2023 | 0.70 | 0.65, 0.74 | 0.02 | 56% |  |
| **Random effects model** | **0.70** | **0.66, 0.74** | **0.02** | **53%** |  |
| **clinical pregnancy (3 m vs 12 m)** | | | | | |
| Costa et al., 2025 | 0.72 | 0.61, 0.84 | < 0.00001 | 87% |  |
| Cui et al., 2021 | 0.66 | 0.61, 0.72 | 0.11 | 36% |  |
| Guo et al., 2025 | 0.72 | 0.62, 0.85 | < 0.00001 | 86% |  |
| Hu et al., 2022 | 0.72 | 0.62, 0.84 | < 0.00001 | 87% |  |
| Li et al., 2016 | 0.70 | 0.60, 0.82 | < 0.00001 | 87% |  |
| Liang et al., 2024 | 0.71 | 0.61, 0.84 | < 0.00001 | 87% |  |
| Ma et al., 2023 | 0.71 | 0.60, 0.83 | < 0.00001 | 87% |  |
| Mao et al., 2022 | 0.72 | 0.60, 0.85 | < 0.00001 | 87% |  |
| Wirleitner et al., 2013 | 0.70 | 0.60, 0.81 | < 0.00001 | 87% |  |
| Zhang et al., 2021 | 0.72 | 0.62, 0.85 | < 0.00001 | 87% |  |
| Zhao et al., 2026 | 0.73 | 0.63, 0.85 | < 0.00001 | 85% |  |
| Zheng et al., 2023 | 0.72 | 0.61, 0.84 | < 0.00001 | 87% |  |
| **Random effects model** | **0.71** | **0.61, 0.82** | **< 0.00001** | **86%** |  |
| **clinical pregnancy (3 m vs 2 y)** | | | | | |
| Li et al., 2016 | 0.76 | 0.64, 0.91 | 0.05 | 61% |  |
| Ma et al., 2023 | 0.76 | 0.61, 0.93 | 0.07 | 57% |  |
| Mao et al., 2022 | 0.82 | 0.62, 1.09 | 0.04 | 65% |  |
| Wirleitner et al., 2013 | 0.73 | 0.64, 0.84 | 0.19 | 37% |  |
| Zheng et al., 2023 | 0.82 | 0.69, 0.98 | 0.23 | 30% |  |
| **Random effects model** | **0.77** | **0.65, 0.91** | **0.08** | **53%** |  |
| **clinical pregnancy (6 m)** | | | | | |
| Costa et al., 2025 | 0.84 | 0.71, 0.99 | < 0.00001 | 95% |  |
| Guo et al., 2025 | 0.85 | 0.72, 1.01 | < 0.00001 | 94% |  |
| Hu et al., 2022 | 0.85 | 0.71, 1.00 | < 0.00001 | 95% |  |
| Lee et al., 2021 | 0.82 | 0.70, 0.97 | < 0.00001 | 95% |  |
| Li et al., 2016 | 0.83 | 0.70, 0.97 | < 0.00001 | 95% |  |
| Li et al., 2023 | 0.82 | 0.70, 0.97 | < 0.00001 | 95% |  |
| Liang et al., 2024 | 0.84 | 0.70, 0.99 | < 0.00001 | 95% |  |
| Ma et al., 2023 | 0.84 | 0.71, 0.99 | < 0.00001 | 95% |  |
| Mao et al., 2022 | 0.85 | 0.71, 1.01 | < 0.00001 | 94% |  |
| Wirleitner et al., 2013 | 0.81 | 0.69, 0.95 | < 0.00001 | 95% |  |
| Zhang et al., 2021 | 0.86 | 0.73, 1.01 | < 0.00001 | 94% |  |
| Zhao et al., 2026 | 0.77 | 0.71, 0.84 | < 0.00001 | 78% |  |
| Zheng et al., 2023 | 0.84 | 0.71, 1.00 | < 0.00001 | 95% |  |
| **Random effects model** | **0.83** | **0.71, 0.97** | **< 0.00001** | **94%** |  |
| **clinical pregnancy (6 m vs 12 m)** | | | | | |
| Costa et al., 2025 | 0.79 | 0.70, 0.89 | < 0.00001 | 78% |  |
| Guo et al., 2025 | 0.81 | 0.72, 0.90 | 0.0002 | 69% |  |
| Hu et al., 2022 | 0.80 | 0.71, 0.91 | < 0.00001 | 77% |  |
| Lee et al., 2021 | 0.77 | 0.68, 0.86 | < 0.00001 | 74% |  |
| Li et al., 2016 | 0.78 | 0.69, 0.88 | < 0.00001 | 77% |  |
| Li et al., 2023 | 0.77 | 0.69, 0.87 | < 0.00001 | 77% |  |
| Liang et al., 2024 | 0.79 | 0.70, 0.90 | < 0.00001 | 78% |  |
| Ma et al., 2023 | 0.78 | 0.69, 0.89 | < 0.00001 | 77% |  |
| Mao et al., 2022 | 0.80 | 0.69, 0.91 | < 0.00001 | 78% |  |
| Wirleitner et al., 2013 | 0.76 | 0.68, 0.85 | < 0.00001 | 72% |  |
| Zhang et al., 2021 | 0.80 | 0.71, 0.91 | < 0.00001 | 76% |  |
| Zhao et al., 2026 | 0.76 | 0.68, 0.85 | < 0.00001 | 72% |  |
| Zheng et al., 2023 | 0.80 | 0.70, 0.91 | < 0.00001 | 78% |  |
| **Random effects model** | **0.79** | **0.70, 0.88** | **< 0.00001** | **76%** |  |
| **clinical pregnancy (6 m vs 2 y)** | | | | | |
| Lee et al., 2021 | 0.88 | 0.70, 1.12 | 0.001 | 77% |  |
| Li et al., 2016 | 0.87 | 0.71, 1.08 | 0.001 | 78% |  |
| Ma et al., 2023 | 0.90 | 0.70, 1.15 | 0.001 | 78% |  |
| Mao et al., 2022 | 0.94 | 0.70, 1.25 | 0.001 | 78% |  |
| Wirleitner et al., 2013 | 0.80 | 0.69, 0.93 | 0.09 | 50% |  |
| Zheng et al., 2023 | 0.96 | 0.78, 1.17 | 0.04 | 61% |  |
| **Random effects model** | **0.89** | **0.72, 1.08** | **0.002** | **73%** |  |
| **clinical pregnancy (12 m)** | | | | | |
| Costa et al., 2025 | 0.80 | 0.72, 0.89 | < 0.00001 | 86% |  |
| Cui et al., 2021 | 0.77 | 0.71, 0.84 | < 0.00001 | 76% |  |
| Guo et al., 2025 | 0.81 | 073, 0.90 | < 0.00001 | 84% |  |
| Hu et al., 2022 | 0.81 | 0.73, 0.90 | < 0.00001 | 86% |  |
| Lee et al., 2021 | 0.79 | 0.71, 0.87 | < 0.00001 | 85% |  |
| Li et al., 2016 | 0.79 | 0.72, 0.88 | < 0.00001 | 86% |  |
| Li et al., 2023 | 0.79 | 0.72, 0.88 | < 0.00001 | 86% |  |
| Liang et al., 2024 | 0.80 | 0.73, 0.89 | < 0.00001 | 86% |  |
| Lin et al., 2021 | 0.80 | 0.72, 0.89 | < 0.00001 | 86% |  |
| Ma et al., 2023 | 0.80 | 0.72, 0.88 | < 0.00001 | 86% |  |
| Mao et al., 2022 | 0.80 | 0.72, 0.90 | < 0.00001 | 86% |  |
| Shi et al., 2022 | 0.79 | 0.71, 0.87 | < 0.00001 | 85% |  |
| Wang et al., 2024 | 0.81 | 0.72, 0.91 | < 0.00001 | 85% |  |
| Wirleitner et al., 2013 | 0.78 | 0.71, 0.86 | < 0.00001 | 85% |  |
| Zhang et al., 2021 | 0.81 | 0.73, 0.90 | < 0.00001 | 85% |  |
| Zhao et al., 2026 | 0.82 | 0.75, 0.90 | < 0.00001 | 84% |  |
| Zheng et al., 2023 | 0.81 | 0.73, 0.90 | < 0.00001 | 86% |  |
| **Random effects model** | **0.80** | **0.73, 0.88** | **< 0.00001** | **85%** |  |
| **clinical pregnancy (12 m vs 2 y)** | | | | | |
| Lee et al., 2021 | 0.91 | 0.72, 1.16 | 0.0007 | 79% |  |
| Li et al., 2016 | 0.90 | 0.72, 1.12 | 0.0006 | 79% |  |
| Ma et al., 2023 | 0.92 | 0.72, 1.19 | 0.0006 | 80% |  |
| Mao et al., 2022 | 0.96 | 0.72, 1.29 | 0.0004 | 80% |  |
| Wirleitner et al., 2013 | 0.83 | 0.70, 0.97 | 0.05 | 57% |  |
| Zheng et al., 2023 | 0.99 | 0.81, 1.21 | 0.04 | 61% |  |
| **Random effects model** | **0.91** | **0.74, 1.13** | **0.001** | **75%** |  |
| **clinical pregnancy (2 y)** | | | | | |
| Lee et al., 2021 | 0.94 | 0.76, 1.17 | 0.0003 | 78% |  |
| Li et al., 2016 | 0.93 | 0.76, 1.13 | 0.0004 | 78% |  |
| Liu et al., 2014 | 0.92 | 0.75, 1.13 | 0.0008 | 76% |  |
| Ma et al., 2023 | 0.94 | 0.75, 1.18 | 0.0004 | 78% |  |
| Mao et al., 2022 | 0.98 | 0.76, 1.25 | 0.0003 | 78% |  |
| Wirleitner et al., 2013 | 0.86 | 0.74, 1.01 | 0.03 | 61% |  |
| Zheng et al., 2023 | 1.00 | 0.84, 1.18 | 0.05 | 55% |  |
| **Random effects model** | **0.94** | **0.78, 1.13** | **0.0007** | **74%** |  |
| **live birth (3 m)** | | | | | |
| Cui et al., 2021 | 0.89 | 0.83, 0.95 | <0.00001 | 85% |  |
| Guo et al., 2025 | 0.90 | 0.84, 0.96 | <0.00001 | 85% |  |
| Hu et al., 2022 | 0.89 | 0.83, 0.96 | <0.00001 | 86% |  |
| Li et al., 2016 | 0.90 | 0.84, 0.96 | <0.00001 | 85% |  |
| Liang et al., 2024 | 0.89 | 0.83, 0.96 | <0.00001 | 86% |  |
| Ma et al., 2023 | 0.90 | 0.84, 0.96 | <0.00001 | 85% |  |
| Mao et al., 2022 | 0.90 | 0.83, 0.97 | <0.00001 | 85% |  |
| Wirleitner et al., 2013 | 0.89 | 0.84, 0.96 | <0.00001 | 86% |  |
| Zhang et al., 2021 | 0.92 | 0.87, 0.97 | <0.00001 | 74% |  |
| Zhao et al., 2026 | 0.88 | 0.84, 0.92 | 0.003 | 64% |  |
| Zheng et al., 2023 | 0.90 | 0.84, 0.96 | <0.00001 | 85% |  |
| **Random effects model** | **0.90** | **0.84, 0.95** | **<0.00001** | **84%** |  |
| **live birth (3 m vs 6 m)** | | | | | |
| Guo et al., 2025 | 0.75 | 0.69, 0.82 | 0.0006 | 71% |  |
| Hu et al., 2022 | 0.75 | 0.68, 0.81 | 0.0003 | 73% |  |
| Li et al., 2016 | 0.74 | 0.69, 0.81 | 0.0002 | 73% |  |
| Liang et al., 2024 | 0.74 | 0.67, 0.80 | 0.0004 | 72% |  |
| Ma et al., 2023 | 0.74 | 0.68, 0.80 | 0.0003 | 73% |  |
| Mao et al., 2022 | 0.73 | 0.67, 0.78 | 0.01 | 59% |  |
| Wirleitner et al., 2013 | 0.74 | 0.68, 0.80 | 0.0004 | 72% |  |
| Zhang et al., 2021 | 0.76 | 0.72, 0.81 | 0.13 | 36% |  |
| Zhao et al., 2026 | 0.74 | 0.68, 0.82 | 0.0002 | 73% |  |
| Zheng et al., 2023 | 0.74 | 0.67, 0.80 | 0.0003 | 72% |  |
| **Random effects model** | **0.74** | **0.69, 0.80** | **0.0005** | **70%** |  |
| **live birth (3 m vs 12 m)** | | | | | |
| Cui et al., 2021 | 0.69 | 0.61, 0.78 | 0.0005 | 70% |  |
| Guo et al., 2025 | 0.74 | 0.63, 0.86 | <0.00001 | 82% |  |
| Hu et al., 2022 | 0.73 | 0.63, 0.86 | <0.00001 | 85% |  |
| Li et al., 2016 | 0.73 | 0.62, 0.84 | <0.00001 | 85% |  |
| Liang et al., 2024 | 0.73 | 0.62, 0.85 | <0.00001 | 85% |  |
| Ma et al., 2023 | 0.72 | 0.61, 0.84 | <0.00001 | 85% |  |
| Mao et al., 2022 | 0.71 | 0.60, 0.84 | <0.00001 | 84% |  |
| Wirleitner et al., 2013 | 0.71 | 0.61, 0.82 | <0.00001 | 85% |  |
| Zhang et al., 2021 | 0.75 | 0.64, 0.86 | <0.00001 | 83% |  |
| Zhao et al., 2026 | 0.75 | 0.65, 0.86 | <0.00001 | 82% |  |
| Zheng et al., 2023 | 0.72 | 0.61, 0.85 | <0.00001 | 85% |  |
| **Random effects model** | **0.72** | **0.63, 0.84** | **<0.00001** | **83%** |  |
| **live birth (6 m)** | | | | | |
| Guo et al., 2025 | 0.85 | 0.76, 0.95 | <0.00001 | 86% |  |
| Hu et al., 2022 | 0.84 | 0.74, 0.95 | <0.00001 | 89% |  |
| Lee et al., 2021 | 0.83 | 0.73, 0.93 | <0.00001 | 89% |  |
| Li et al., 2016 | 0.83 | 0.73, 0.93 | <0.00001 | 89% |  |
| Li et al., 2023 | 0.82 | 0.73, 0.92 | <0.00001 | 89% |  |
| Liang et al., 2024 | 0.83 | 0.73, 0.95 | <0.00001 | 89% |  |
| Ma et al., 2023 | 0.83 | 0.74, 0.94 | <0.00001 | 89% |  |
| Mao et al., 2022 | 0.83 | 0.73, 0.95 | <0.00001 | 89% |  |
| Wirleitner et al., 2013 | 0.81 | 0.72, 0.91 | <0.00001 | 89% |  |
| Zhang et al., 2021 | 0.85 | 0.76, 0.95 | <0.00001 | 86% |  |
| Zhao et al., 2026 | 0.79 | 0.73, 0.87 | <0.00001 | 77% |  |
| Zheng et al., 2023 | 0.83 | 0.74, 0.94 | <0.00001 | 89% |  |
| **Random effects model** | **0.83** | **0.74, 0.93** | **<0.00001** | **88%** |  |
| **live birth (6 m vs 12 m)** | | | | | |
| Guo et al., 2025 | 0.80 | 0.72, 0.89 | 0.010 | 57% |  |
| Hu et al., 2022 | 0.79 | 0.70, 0.89 | <0.0001 | 73% |  |
| Lee et al., 2021 | 0.76 | 0.68, 0.86 | <0.0001 | 72% |  |
| Li et al., 2016 | 0.77 | 0.69, 0.87 | <0.0001 | 74% |  |
| Li et al., 2023 | 0.77 | 0.68, 0.86 | <0.0001 | 73% |  |
| Liang et al., 2024 | 0.79 | 0.69, 0.89 | <0.0001 | 74% |  |
| Ma et al., 2023 | 0.77 | 0.68, 0.88 | <0.0001 | 74% |  |
| Mao et al., 2022 | 0.77 | 0.68, 0.87 | 0.0003 | 70% |  |
| Wirleitner et al., 2013 | 0.75 | 0.68, 0.84 | 0.0006 | 68% |  |
| Zhang et al., 2021 | 0.80 | 0.71, 0.89 | 0.0003 | 70% |  |
| Zhao et al., 2026 | 0.78 | 0.69, 0.88 | <0.0001 | 74% |  |
| Zheng et al., 2023 | 0.78 | 0.68, 0.88 | <0.0001 | 74% |  |
| **Random effects model** | **0.78** | **0.69, 0.87** | **<0.0001** | **72%** |  |
| **live birth (12 m)** | | | | | |
| Cui et al., 2021 | 0.77 | 0.71, 0.84 | <0.00001 | 73% |  |
| Guo et al., 2025 | 0.81 | 0.73, 0.89 | <0.00001 | 80% |  |
| Hu et al., 2022 | 0.80 | 0.73, 0.88 | <0.00001 | 82% |  |
| Lee et al., 2021 | 0.78 | 0.71, 0.86 | <0.00001 | 82% |  |
| Li et al., 2016 | 0.79 | 0.72, 0.87 | <0.00001 | 83% |  |
| Li et al., 2023 | 0.79 | 0.72, 0.87 | <0.00001 | 82% |  |
| Liang et al., 2024 | 0.80 | 0.73, 0.88 | <0.00001 | 83% |  |
| Lin et al., 2021 | 0.79 | 0.72, 0.88 | <0.00001 | 83% |  |
| Ma et al., 2023 | 0.79 | 0.72, 0.87 | <0.00001 | 83% |  |
| Mao et al., 2022 | 0.79 | 0.71, 0.87 | <0.00001 | 82% |  |
| Shi et al., 2022 | 0.79 | 0.72, 0.87 | <0.00001 | 82% |  |
| Wang et al., 2024 | 0.80 | 0.72, 0.89 | <0.00001 | 82% |  |
| Wirleitner et al., 2013 | 0.78 | 0.71, 0.85 | <0.00001 | 81% |  |
| Zhang et al., 2021 | 0.81 | 0.74, 0.89 | <0.00001 | 81% |  |
| Zhao et al., 2026 | 0.81 | 0.75, 0.89 | <0.00001 | 79% |  |
| Zheng et al., 2023 | 0.79 | 0.72, 0.88 | <0.00001 | 83% |  |
| **Random effects model** | **0.79** | **0.72, 0.87** | **<0.00001** | **81%** |  |
| **live birth (2 y)** | | | | | |
| Lee et al., 2021 | 0.94 | 0.81, 1.08 | 0.003 | 70% |  |
| Li et al., 2016 | 0.93 | 0.81, 1.06 | 0.002 | 71% |  |
| Liu et al., 2014 | 0.93 | 0.81, 1.08 | 0.002 | 70% |  |
| Ma et al., 2023 | 0.93 | 0.81, 1.08 | 0.003 | 70% |  |
| Mao et al., 2022 | 0.95 | 0.83, 1.09 | 0.02 | 61% |  |
| Wirleitner et al., 2013 | 0.90 | 0.80, 1.03 | 0.007 | 66% |  |
| Zhan et al., 2024 | 0.88 | 0.79, 0.99 | 0.21 | 28% |  |
| Zheng et al., 2023 | 0.97 | 0.87, 1.09 | 0.08 | 47% |  |
| **Random effects model** | **0.93** | **0.82, 1.06** | **0.005** | **66%** |  |
| **biochemical pregnancy (3 m)** | | | | | |
| Hu et al., 2022 | 079 | 0.71, 0.89 | < 0.00001 | 89% |  |
| Liang et al., 2024 | 0.80 | 0.70, 0.90 | < 0.00001 | 92% |  |
| Ma et al., 2023 | 0.82 | 0.72, 0.93 | < 0.00001 | 94% |  |
| Mao et al., 2022 | 0.85 | 0.78, 0.93 | 0.0005 | 77% |  |
| Wirleitner et al., 2013 | 0.81 | 0.72, 0.92 | < 0.00001 | 94% |  |
| Zhang et al., 2021 | 0.82 | 0.71, 0.95 | < 0.00001 | 94% |  |
| Zheng et al., 2023 | 0.83 | 0.72, 0.95 | < 0.00001 | 94% |  |
| **Random effects model** | **0.82** | **0.73, 0.92** | **< 0.00001** | **92%** |  |
| **biochemical pregnancy (3 m vs 6 m)** | | | | | |
| Hu et al., 2022 | 0.70 | 0.62, 0.79 | 0.0002 | 80% |  |
| Liang et al., 2024 | 0.67 | 0.61, 0.73 | 0.03 | 60% |  |
| Ma et al., 2023 | 0.69 | 0.62, 0.77 | 0.0002 | 79% |  |
| Mao et al., 2022 | 0.72 | 0.64, 0.81 | 0.004 | 71% |  |
| Wirleitner et al., 2013 | 0.69 | 0.62, 0.76 | 0.0003 | 78% |  |
| Zhang et al., 2021 | 0.72 | 0.64, 0.81 | 0.0006 | 77% |  |
| Zheng et al., 2023 | 0.71 | 0.63, 0.80 | < 0.0001 | 81% |  |
| **Random effects model** | **0.70** | **0.63, 0.77** | **0.0002** | **77%** |  |
| **biochemical pregnancy (3 m vs 12 m)** | | | | | |
| Hu et al., 2022 | 0.74 | 0.67, 0.81 | 0.03 | 61% |  |
| Liang et al., 2024 | 0.72 | 0.64, 0.81 | 0.006 | 69% |  |
| Ma et al., 2023 | 0.71 | 0.64, 0.80 | 0.007 | 69% |  |
| Mao et al., 2022 | 0.73 | 0.65, 0.83 | 0.03 | 59% |  |
| Wirleitner et al., 2013 | 0.71 | 0.65, 0.78 | 0.02 | 63% |  |
| Zhang et al., 2021 | 0.70 | 0.63, 0.77 | 0.14 | 39% |  |
| Zheng et al., 2023 | 0.74 | 0.66, 0.82 | 0.03 | 61% |  |
| **Random effects model** | 0.72 | 0.65, 0.80 | 0.01 | 63% |  |
| **biochemical pregnancy (3 m vs 2 y)** | | | | | |
| Ma et al., 2023 | 0.72 | 0.56, 0.91 | 0.04 | 69% |  |
| Mao et al., 2022 | 0.78 | 0.56, 1.08 | 0.02 | 76% |  |
| Wirleitner et al., 2013 | 0.70 | 0.59, 0.83 | 0.08 | 61% |  |
| Zheng et al., 2023 | 0.80 | 0.66, 0.96 | 0.20 | 37% |  |
| **Random effects model** | **0.74** | **0.61, 0.90** | **0.04** | **64%** |  |
| **biochemical pregnancy (6 m)** | | | | | |
| Hu et al., 2022 | 0.75 | 0.66, 0.85 | < 0.0001 | 82% |  |
| Liang et al., 2024 | 0.72 | 0.65, 0.80 | 0.002 | 73% |  |
| Ma et al., 2023 | 0.73 | 0.65, 0.82 | < 0.0001 | 81% |  |
| Mao et al., 2022 | 0.76 | 0.67, 0.86 | 0.0002 | 79% |  |
| Wirleitner et al., 2013 | 0.71 | 0.65, 0.77 | 0.006 | 70% |  |
| Zhang et al., 2021 | 0.76 | 0.68, 0.86 | 0.0003 | 79% |  |
| Zheng et al., 2023 | 0.75 | 0.67, 0.85 | < 0.0001 | 82% |  |
| **Random effects model** | **0.74** | **0.67, 0.82** | **< 0.0001** | **79%** |  |
| **biochemical pregnancy (6 m vs 12 m)** | | | | | |
| Hu et al., 2022 | 0.80 | 0.71, 0.90 | 0.001 | 75% |  |
| Liang et al., 2024 | 0.78 | 0.68, 0.89 | 0.0001 | 80% |  |
| Ma et al., 2023 | 0.77 | 0.67, 0.88 | 0.0001 | 81% |  |
| Mao et al., 2022 | 0.78 | 0.67, 0.91 | 0.0003 | 78% |  |
| Wirleitner et al., 2013 | 0.74 | 0.67, 0.82 | 0.01 | 67% |  |
| Zhang et al., 2021 | 0.76 | 0.65, 0.89 | 0.002 | 74% |  |
| Zheng et al., 2023 | 0.79 | 0.70, 0.90 | 0.0009 | 76% |  |
| **Random effects model** | **0.77** | **0.68, 0.87** | **0.0002** | **77%** |  |
| **biochemical pregnancy (6 m vs 2 y)** | | | | | |
| Ma et al., 2023 | 0.84 | 0.60, 1.17 | 0.0006 | 87% |  |
| Mao et al., 2022 | 0.88 | 0.58, 1.34 | 0.0004 | 87% |  |
| Wirleitner et al., 2013 | 0.74 | 0.61, 0.89 | 0.04 | 68% |  |
| Zheng et al., 2023 | 0.93 | 0.71, 1.23 | 0.02 | 74% |  |
| **Random effects model** | 0.83 | 0.65, 1.07 | 0.001 | 81% |  |
| **biochemical pregnancy (12 m)** | | | | | |
| Hu et al., 2022 | 0.78 | 0.72, 0.85 | 0.001 | 73% |  |
| Liang et al., 2024 | 0.77 | 0.70, 0.84 | 0.0003 | 77% |  |
| Ma et al., 2023 | 0.76 | 0.69, 0.83 | 0.0004 | 76% |  |
| Mao et al., 2022 | 0.77 | 0.70, 0.85 | 0.0003 | 77% |  |
| Wang et al., 2024 | 0.78 | 0.70, 0.87 | 0.001 | 73% |  |
| Wirleitner et al., 2013 | 0.75 | 0.70, 0.79 | 0.12 | 41% |  |
| Zhang et al., 2021 | 0.77 | 0.69, 0.85 | 0.001 | 73% |  |
| Zheng et al., 2023 | 0.78 | 0.71, 0.85 | 0.0006 | 75% |  |
| **Random effects model** | **0.77** | **0.70, 0.83** | **0.0006** | **73%** |  |
| **biochemical pregnancy (12 m vs 2 y)** | | | | | |
| Ma et al., 2023 | 0.87 | 0.61, 1.23 | 0.0002 | 88% |  |
| Mao et al., 2022 | 0.91 | 0.59, 1.40 | 0.0002 | 89% |  |
| Wirleitner et al., 2013 | 0.76 | 0.62, 0.93 | 0.03 | 71% |  |
| Zheng et al., 2023 | 0.97 | 0.73, 1.28 | 0.02 | 75% |  |
| **Random effects model** | **0.86** | **0.66, 1.12** | **0.0005** | **83%** |  |
| **biochemical pregnancy (2 y)** | | | | | |
| Ma et al., 2023 | 0.85 | 0.61, 1.19 | 0.0005 | 87% |  |
| Mao et al., 2022 | 0.89 | 0.59, 1.35 | 0.0003 | 87% |  |
| Wirleitner et al., 2013 | 0.77 | 0.62, 094 | 0.02 | 73% |  |
| Zheng et al., 2023 | 0.95 | 0.74, 1.22 | 0.04 | 69% |  |
| **Random effects model** | **0.86** | **0.67, 1.10** | **0.001** | **82%** |  |
| **embryo implantation rate (3m vs 6 m)** | | | | | |
| Li et al., 2016 | 0.78 | 0.46, 1.33 | 0.006 | 87% |  |
| Mao et al., 2022 | 0.81 | 0.49, 1.34 | 0.04 | 77% |  |
| Wirleitner et al., 2013 | 0.62 | 0.58, 0.65 | 0.84 | 0% |  |
| **Random effects model** | **0.71** | **0.54, 0.94** | **0.02** | **74%** |  |
| **embryo implantation rate (3m vs 12 m)** | | | | | |
| Li et al., 2016 | 0.89 | 0.50, 1.60 | 0.004 | 88% |  |
| Mao et al., 2022 | 0.87 | 0.43, 1.74 | 0.009 | 85% |  |
| Wirleitner et al., 2013 | 0.68 | 0.62, 0.74 | 0.56 | 0% |  |
| **Random effects model** | **0.78** | **0.56, 1.09** | **0.01** | **78%** |  |
| **embryo implantation rate (3m vs 2 y)** | | | | | |
| Li et al., 2016 | 0.97 | 0.51, 1.83 | 0.004 | 88% |  |
| Mao et al., 2022 | 0.96 | 0.45, 2.02 | 0.02 | 81% |  |
| Wirleitner et al., 2013 | 0.72 | 0.64, 0.81 | 0.67 | 0% |  |
| **Random effects model** | **0.85** | **0.57, 1.29** | **0.01** | **77%** |  |
| **embryo implantation rate (6 m)** | | | | | |
| Lee et al., 2021 | 0.85 | 0.64, 1.11 | < 0.00001 | 90% |  |
| Li et al., 2016 | 0.94 | 0.69, 1.28 | < 0.00001 | 94% |  |
| Li et al., 2023 | 0.86 | 0.64, 1.14 | < 0.00001 | 93% |  |
| Mao et al., 2022 | 0.95 | 0.76, 1.19 | 0.002 | 79% |  |
| Wirleitner et al., 2013 | 0.82 | 0.64, 1.06 | < 0.00001 | 92% |  |
| **Random effects model** | **0.88** | **0.69, 1.13** | **< 0.00001** | **92%** |  |
| **embryo implantation rate (6m vs 12 m)** | | | | | |
| Lee et al., 2021 | 0.90 | 0.67, 1.20 | < 0.00001 | 87% |  |
| Li et al., 2016 | 1.01 | 0.76, 1.35 | < 0.00001 | 90% |  |
| Li et al., 2023 | 0.92 | 0.68, 1.24 | < 0.00001 | 90% |  |
| Mao et al., 2022 | 1.00 | 0.75, 132 | 0.002 | 80% |  |
| Wirleitner et al., 2013 | 0.85 | 0.68, 1.07 | 0.003 | 84% |  |
| **Random effects model** | **0.93** | **0.73, 1.20** | **< 0.00001** | **88%** |  |
| **embryo implantation rate (6m vs 2 y)** | | | | | |
| Lee et al., 2021 | 0.95 | 0.59, 1.53 | 0.0004 | 87% |  |
| Li et al., 2016 | 1.02 | 0.72, 1.45 | 0.0004 | 87% |  |
| Mao et al., 2022 | 1.03 | 0.67, 1.58 | 0.006 | 80% |  |
| Wirleitner et al., 2013 | 0.82 | 0.72, 0.94 | 0.27 | 23% |  |
| **Random effects model** | **0.95** | **0.71, 1.27** | **0.0009** | **82%** |  |
| **embryo implantation rate (12 m)** | | | | | |
| Lee et al., 2021 | 0.94 | 0.76, 1.16 | < 0.00001 | 85% |  |
| Li et al., 2016 | 1.01 | 0.82, 1.25 | < 0.00001 | 87% |  |
| Li et al., 2023 | 0.96 | 0.77, 1.19 | < 0.00001 | 88% |  |
| Mao et al., 2022 | 1.02 | 0.85, 1.23 | 0.006 | 72% |  |
| Shi et al., 2022 | 0.97 | 0.77, 1.23 | < 0.00001 | 87% |  |
| Wirleitner et al., 2013 | 0.90 | 0.76, 1.05 | 0.001 | 78% |  |
| **Random effects model** | **0.96** | **0.80, 1.16** | **< 0.00001** | **85%** |  |
| **embryo implantation rate (12m vs 2 y)** | | | | | |
| Lee et al., 2021 | 1.02 | 0.63, 1.64 | 0.0002 | 88% |  |
| Li et al., 2016 | 1.06 | 0.75, 1.51 | 0.0002 | 88% |  |
| Mao et al., 2022 | 1.08 | 0.71, 1.65 | 0.005 | 81% |  |
| Wirleitner et al., 2013 | 0.84 | 0.76, 0.93 | 0.43 | 0% |  |
| **Random effects model** | **0.99** | **0.74, 1.33** | **0.0006** | **83%** |  |
| **embryo implantation rate (12m vs 3 y)** | | | | | |
| Li et al., 2023 | 1.20 | 0.71, 2.03 | 0.04 | 76% |  |
| Shi et al., 2022 | 1.37 | 1.02, 1.84 | 0.34 | 0% |  |
| Wirleitner et al., 2013 | 1.03 | 0.83, 1.28 | 0.24 | 27% |  |
| **Random effects model** | **1.17** | **0.87, 1.58** | **0.09** | **59%** |  |
| **embryo implantation rate (2 y)** | | | | | |
| Lee et al., 2021 | 1.04 | 0.86, 1.25 | < 0.00001 | 84% |  |
| Li et al., 2016 | 1.04 | 0.88, 1.23 | < 0.00001 | 84% |  |
| Liu et al., 2014 | 1.00 | 0.83, 1.19 | < 0.00001 | 84% |  |
| Mao et al., 2022 | 1.08 | 0.92, 1.25 | 0.04 | 60% |  |
| Wirleitner et al., 2013 | 0.95 | 0.82, 1.11 | 0.001 | 77% |  |
| Zhan et al., 2024 | 1.01 | 0.81, 1.27 | 0.001 | 78% |  |
| **Random effects model** | **1.02** | **0.87, 1.19** | **0.001** | **80%** |  |
| **congenital malformation (2 y)** | | | | | |
| Lee et al., 2021 | 0.94 | 0.42, 2.13 | 0.10 | 63.9% |  |
| Li et al., 2016 | 0.88 | 0.41, 1.87 | 0.11 | 60.8% |  |
| Mao et al., 2022 | 1.38 | 0.39, 4.92 | 0.02 | 80.1% |  |
| Wirleitner et al., 2013 | 1.01 | 0.43, 2.38 | 0.08 | 66.7% |  |
| Zhan et al., 2024 | 1.53 | 0.68, 3.45 | 0.64 | 0% |  |
| **Random effects model** | **1.06** | **0.48, 2.30** | **0.06** | **71.2%** |  |
| **male children (3 m)** | | | | | |
| Cui et al., 2021 | 0.97 | 0.90, 1.05 | 0.36 | 1.6% |  |
| Li et al., 2016 | 0.99 | 0.92, 1.07 | 0.12 | 52.2% |  |
| Li et al., 2020 | 1.00 | 0.91, 1.10 | 0.02 | 74.4% |  |
| Mao et al., 2022 | 1.02 | 0.95, 1.09 | 0.65 | 0% |  |
| Xu et al., 2022 | 0.96 | 0.90, 1.01 | 0.13 | 57.4% |  |
| **Random effects model** | **0.99** | **0.92, 1.06** | **0.12** | **52.2%** |  |
| **male children (12 m)** | | | | | |
| Cui et al., 2021 | 0.95 | 0.88, 1.01 | 0.32 | 12.7% |  |
| Lee et al., 2021 | 0.96 | 0.90, 1.03 | 0.14 | 48.7% |  |
| Li et al., 2016 | 0.96 | 0.90, 1.02 | 0.13 | 50.2% |  |
| Li et al., 2020 | 0.96 | 0.90, 1.02 | 0.13 | 50.3% |  |
| Mao et al., 2022 | 0.97 | 0.90, 1.04 | 0.15 | 47.7% |  |
| Shi et al., 2022 | 0.97 | 0.91, 1.03 | 0.16 | 45.1% |  |
| Wang et al., 2024 | 0.97 | 0.89, 1.06 | 0.11 | 55.2% |  |
| Xu et al., 2022 | 0.95 | 0.89, 1.02 | 0.13 | 56.7% |  |
| **Random effects model** | **0.96** | **0.90, 1.02** | **0.13** | **50.9%** |  |
| **male children (2 y)** | | | | | |
| Lee et al., 2021 | 0.91 | 0.77, 1.08 | 0.09 | 64.4% |  |
| Li et al., 2016 | 0.90 | 0.77, 1.05 | 0.07 | 69.9% |  |
| Mao et al., 2022 | 0.98 | 0.88, 1.08 | 0.61 | 0% |  |
| Zhan et al., 2024 | 0.81 | 0.68, 0.98 | 0.76 | 0% |  |
| **Random effects model** | **0.92** | **0.81, 1.04** | **0.08** | **66.6%** |  |

1. OR: Odd ratio.
2. m: months.
3. Random effects model was used to calculate statistical values.
4. y: years.
